# Magnetic resonance imaging–based machine learning classification of schizophrenia spectrum disorders: a meta‐analysis

**DOI:** 10.1111/pcn.13736

**Published:** 2024-09-18

**Authors:** Fabio Di Camillo, David Antonio Grimaldi, Giulia Cattarinussi, Annabella Di Giorgio, Clara Locatelli, Adyasha Khuntia, Paolo Enrico, Paolo Brambilla, Nikolaos Koutsouleris, Fabio Sambataro

**Affiliations:** ^1^ Department of Neuroscience (DNS) University of Padova Padua Italy; ^2^ Padova Neuroscience Center University of Padova Padua Italy; ^3^ Department of Psychological Medicine, Institute of Psychiatry, Psychology and Neuroscience King's College London London United Kingdom; ^4^ Department of Mental Health and Addictions ASST Papa Giovanni XXIII Bergamo Italy; ^5^ Department of Psychiatry and Psychotherapy Ludwig‐Maximilian University Munich Germany; ^6^ International Max Planck Research School for Translational Psychiatry (IMPRS‐TP) Munich Germany; ^7^ Max‐Planck‐Institute of Psychiatry Munich Germany; ^8^ Department of Pathophysiology and Transplantation University of Milan Milan Italy; ^9^ Department of Neurosciences and Mental Health Fondazione IRCSS Ca’ Granda Ospedale Maggiore Policlinico Milan Italy; ^10^ Department of Psychiatry Munich University Hospital Munich Germany; ^11^ Department of Psychosis Studies, Institute of Psychiatry, Psychology and Neuroscience King's College London London United Kingdom

**Keywords:** machine learning, classification, schizophrenia, magnetic resonance imaging, meta‐analysis

## Abstract

**Background:**

Recent advances in multivariate pattern recognition have fostered the search for reliable neuroimaging‐based biomarkers in psychiatric conditions, including schizophrenia. These approaches consider the complex pattern of alterations in brain function and structure, overcoming the limitations of traditional univariate methods. To assess the reliability of neuroimaging‐based biomarkers and the contribution of study characteristics in distinguishing individuals with schizophrenia spectrum disorder (SSD) from healthy controls (HCs), we conducted a systematic review of the studies that used multivariate pattern recognition for this objective.

**Methods:**

We systematically searched PubMed, Scopus, and Web of Science for studies on SSD classification using multivariate pattern analysis on magnetic resonance imaging data. We employed a bivariate random‐effects meta‐analytic model to explore the classification of sensitivity (SE) and specificity (SP) across studies while also evaluating the moderator effects of clinical and non‐clinical variables.

**Results:**

A total of 119 studies (with 12,723 patients with SSD and 13,196 HCs) were identified. The meta‐analysis estimated a SE of 79.1% (95% confidence interval [CI], 77.1%–81.0%) and a SP of 80.0% (95% CI, 77.8%–82.0%). In particular, the Positive and Negative Syndrome Scale and the Global Assessment of Functioning scores, age, age of onset, duration of untreated psychosis, deep learning, algorithm type, features selection, and validation methods had significant effects on classification performance.

**Conclusions:**

Multivariate pattern analysis reliably identifies neuroimaging‐based biomarkers of SSD, achieving ∼80% SE and SP. Despite clinical heterogeneity, discernible brain modifications effectively differentiate SSD from HCs. Classification performance depends on patient‐related and methodological factors crucial for the development, validation, and application of prospective models in clinical settings.

Schizophrenia spectrum disorders (SSDs) are a group of heterogeneous disorders characterized by psychotic symptoms, with different severity and uncertain cause.^1^, ^2^, ^3^ In the past decades, neuroimaging techniques have become a promising additional tool for the diagnosis and detection of brain structural and functional abnormalities associated with psychiatric disorders.^4^ A large body of neuroimaging research on SSD is now available, showing a general pattern of abnormalities in subcortical areas, including the hippocampus, amygdala, thalamus, basal ganglia,^5^ and fronto‐parietal regions.^6^ In addition, individuals with first‐episode psychosis and a clinical high risk for psychosis present brain alterations in terms of both structure and function, affecting primarily the prefrontal‐striatal‐thalamic circuit.^7^, ^8^, ^9^ However, in recent years, there has been a growing critique of the classical inferential approach, particularly concerning its ability to ensure reproducibility and replication in research,^10^, ^11^ with replication rates plunging as low as 11% for preclinical studies.^12^ Moreover, even if the research findings are reproducible, they often lack clinical significance.^13^ Large sample sizes may yield statistical significance, but this does not necessarily translate into meaningful differences at the individual level, posing a challenge in translating results at the group level.^14^, ^15^ In addition, traditional statistical techniques may overlook critical interactions^16^, ^17^ and individual differences,^18^ which hinders the discovery of biomarkers and their practical application beyond research settings.^19^ Consequently, the translational impact of these findings in clinical practice has been limited to date, as traditional univariate statistics did not adequately deal with high‐dimensional neuroimaging data and are limited in their ability to identify multiregional patterns of variations, such as those observed in SSD.^20^ To overcome this limitation, in recent years, machine learning (ML) techniques have started to be employed to identify patterns of abnormalities that allow the differentiation of individuals with psychiatric disorders between each other and from healthy controls (HCs).^21^ The potential use of ML in clinical practice is related to the diagnosis, prognosis, and prediction of treatment response at the individual level^22^ and involves the computational determination of methods and parameters to achieve an optimal solution to a problem that would traditionally rely on assumptions, inferences, and manual programming by an experimenter.^23^ A comprehensive understanding of ML methodologies encompasses a thorough exploration of various ML paradigms, including well‐established supervised learning, which relies on labeled data to make predictions,^24^ as well as unsupervised learning for discovering hidden patterns within unannotated data.^25^ Moreover, deep learning (DL), a subset of ML, introduces neural networks with multiple layers to process and learn complex features from data.^26^ This methodology has exhibited exceptional performance in complex tasks, often outperforming conventional ML algorithms.^27^ Notably, in the context of psychiatric disorders, these methods have demonstrated substantial promise, including in discerning nuanced patterns within neuroimaging data.^28^ Furthermore, ML is particularly adaptable to the complex structure of connectomic data derived from functional magnetic resonance imaging (fMRI) and diffusion tensor imaging (DTI) techniques.^21^, ^29^

Several reviews and meta‐analyses on the use of ML for classifying patients with psychiatric conditions such as schizophrenia, major depressive disorder, and psychosis risk syndromes from HCs, showed accuracy rates ranging from 77% to 80% across all algorithms investigated, highlighting how ML could improve current psychiatric practice.^30^, ^31^, ^32^ However, the available ML literature on SSD is affected by significant methodological heterogeneity, such as participant inclusion criteria, ML algorithms, and validation techniques.^33^ To date, it remains uncertain whether a specific gold‐standard workflow exists for various classification models that account for the influence of clinical and demographic variables, and/or optimization of setting parameters based on the specific data analyzed. Clinical (e.g. symptom severity and duration of illness) and demographic factors (e.g. age and sex) can significantly impact brain imaging markers at the individual level and, likely, the performance of ML models.^34^, ^35^ Similarly, methodological choices, including feature selection techniques, data preprocessing methods, and model validation strategies, can greatly affect the robustness and accuracy of classification outcomes.^36^, ^37^, ^38^ This lack of clarity poses challenges to both the interpretability and generalizability of the models, emphasizing the importance of investigating the effect of such variables. These analyses are essential for understanding how different factors may impact the classification performance of ML models and, consequently, contribute to addressing the current challenges in the field. By elucidating these influences, researchers can develop more standardized and reliable workflows that enhance the applicability and clinical utility of ML models in SSD research.

In this context, we performed a systematic review and meta‐analysis to investigate the accuracy of different ML algorithms to distinguish individuals with SSD from HCs based on multimodal MRI markers, such as structural MRI (sMRI), diffusion tensor imaging (DTI), resting‐state fMRI (rs‐fMRI), and task‐based fMRI (task‐fMRI), alone or in combination. Our approach sought to evaluate the reliability of ML algorithms in diagnosing SSD based on multivariate patterns of brain variations detected by MRI. In addition, we aimed to identify individual and methodological factors that could exert the greatest influence on classification performance. By addressing these factors, our study aimed to provide insight into optimizing ML workflows for more accurate and generalizable models in the context of SSD diagnosis.

## Methods

### Article selection and classification

**Fig. 1:**
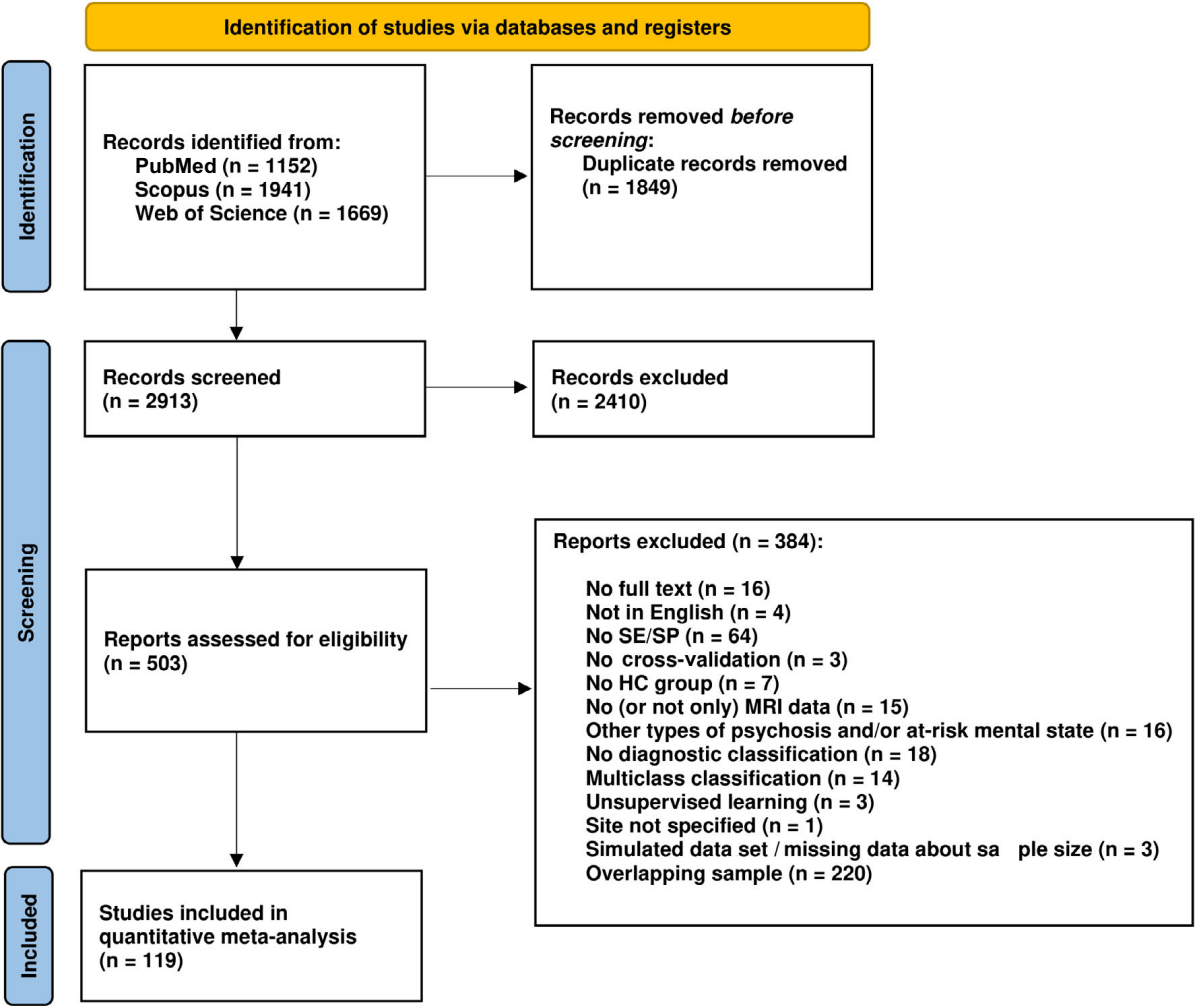
PRISMA (Preferred Reporting Items for Systematic Reviews and Meta‐Analyses) flowchart for the meta‐analysis of imaging articles using machine learning in schizophrenia spectrum disorder. HC, healthy control; MRI, magnetic resonance imaging; SE, sensitivity; SP, specificity.

This systematic review followed a predefined protocol available online (https://osf.io/9dkty) and was performed according to PRISMA (Preferred Reporting Items for Systematic Reviews and Meta‐Analyses) guidelines and flow diagram (Fig. 1).^39^

### Eligibility criteria

Articles met the inclusion criteria if: (i) they applied a supervised ML model to binary classify clinical status using neuroimaging data (sMRI, rs‐fMRI, task‐fMRI, DTI, or a combination of these) as features; (ii) they reported outcomes measures including true positives (TPs), true negatives (TNs), false positives (FPs), and false negatives (FNs) or data that allowed for their calculation; (iii) they implemented some form of internal validation or external data set validation; and (iv) patients met a diagnosis for an SSD according to the *DSM* or *ICD*.

In the event of multiple studies based on the same sample or with a large overlap between samples (>20%), we included only the study with the largest sample size; when the sample sizes were the same between studies, we selected only the study that achieved the best classification accuracy estimated by balanced accuracy (BAC). However, studies that used the same sample were included if their analyses were based on different MRI modalities (e.g. sMRI and rs‐fMRI) to account for the complementary information provided by different imaging techniques.

We excluded non peer‐reviewed publications, intervention‐based study designs, analyses classifying between multiple diagnostic groups other than patients with SSD and HCs, articles in a language different from English, articles with unverified sample provenance to prevent bias from overlapping samples, analyses that used non‐MRI features (alone or in combination with MRI features), studies that used a simulated or synthetically created data set, and analyses that included patients affected by non‐SSD psychosis, such as affective psychosis, substance‐induced psychosis, and psychosis associated with borderline personality disorder. For studies with insufficient details, the authors were contacted to provide additional information, and when they did not respond, the studies were excluded. In case of potential overlap of samples between studies, the authors were contacted to explain this issue. When they did not respond, the studies were treated as if there was overlap and included according to the criteria described above.

### Search strategy

We conducted a systematic bibliographic search procedure including all possibly eligible articles published from inception until 15 August 2023 on PubMed, Scopus, and Web of Science. Titles, abstracts, and keywords were searched using the following terms: (“machine learning” OR “classification” OR “pattern classification” OR “deep learning” OR “multivariate pattern analysis”) AND (“neuroimaging” OR “magnetic resonance image” OR “brain imaging” OR “structural” OR “MRI” OR “functional MRI” OR “fMRI” OR “DTI” OR “diffusion tensor imaging” OR “connectivity” OR “gray matter” OR “white matter”) AND (“schizophreni*” OR “psychosis” OR “psychotic”). After the removal of duplicates, the database was split into two halves, and each two couples of authors (F.D.C. and G.C., A.D.G. and G.L.) independently performed a preliminary screening of titles and abstracts, and according to inclusion criteria, a final decision was made on the full‐text. Disagreements of full‐text articles from two of the four independent reviewers were resolved through discussion in the presence of the other two, and the reasons for the exclusion of full texts were collected. All four authors independently extracted the data, and inconsistencies were cross‐checked.

### Primary outcome and data extraction

The main outcome measure was the diagnostic performance of different ML approaches, assessed using sensitivity (SE) (calculated as TP/(TP + FN)) and specificity (SP) (calculated as TN/(TN + FP)). In studies in which multiple analyses were reported based on different algorithms, validation methods, brain features within the same data modality (e.g. white matter volume or gray matter volume for sMRI), or feature engineering methods, we included the best‐performing model (based on BAC) for further analysis. For studies reporting multiple analyses achieving the same BAC, we averaged the values of SE and SP of the models. Additional information including authors' names, year of publication, population characteristics, clinical scales, including the Positive and Negative Syndrome Scale (PANSS)^40^, ^41^ and General Assessment of Functioning (GAF),^42^ neuroimaging data types, classification model characteristics, and classification metrics were extracted. Furthermore, the PANSS positive‐to‐negative ratio was calculated by dividing PANSS positive by PANSS negative scores. This ratio was calculated to take into account brain differences in brain alterations between patients with predominantly positive symptoms compared with those with predominantly negative symptoms.^43^, ^44^ We classified the feature selection methods into five categories^22^: *a priori* (using previous literature), filter (based on statistical measures, often relying on univariate statistical methods such as *t* test or anova), wrappers (estimated from the feature subsets that contribute the most to model performance by training and assessing models externally, often through iterative processes such as recursive feature elimination), embedded (when feature selection is directly integrated into the model training process, optimizing the model by selecting relevant features inherently, e.g. lasso or ridge regression) and multimodal feature selection methods (when using a combination of these methods), and regress for these factors also including the “no feature selection” factor for studies that did not perform any selection of the features prior to classification. We assessed the quality of primary diagnostic accuracy studies using the QUADAS‐2 scale,^45^ which consists of four domains (patient selection, index test, reference standard, flow and timing) (Supplementary Materials, Fig. S20). The quality assessment was performed independently by two authors (F.D.C. and G.C.), and any disagreement was resolved by discussion.

### Data‐analysis

In this analysis, we followed the methodology introduced by Reitsma et al.^46^ This approach combines log‐transformed SE and SP into a single bivariate regression model, while explicitly considering their correlation. We acknowledge that SE and SP may vary between studies due to differences in study populations, sampling errors, and variations in the thresholds used to distinguish patients from HCs. Therefore, we applied a random‐effects model to address heterogeneity between studies. To minimize the effect of sample size on effect estimates, the meta‐analysis measures were weighted by their sample size. The findings of the meta‐analysis are visually presented through forest plots, separately illustrating SE and SP (Figs S1 and S2). Furthermore, in addition to the main analysis, we also conducted two separate meta‐analyses: one stratifying the studies by MRI modality (Figs S3 and S4) and one dividing the studies based on the stage of the disease (chronic and first‐episode, Fig. S5). When a minimum of five studies^47^ investigated a potential confounding factor (e.g. age, sex, duration of the illness, medication, etc.), we evaluated its impact on the results by incorporating it as a moderator variable in the bivariate regression model. To explore the potential presence of publication bias in meta‐analyses of diagnostic accuracies, funnel plots that presented log diagnostic odds ratios (logDOR) were drawn for each study. An asymmetry in the distribution of the studies in the funnel plot estimated using a regression of logDOR weighted by ESS^48^ (Fig. S17) could suggest publication bias. All analyses were performed using version 2.10.13 of the R statistical programming language^49^ with *mada* package.^50^

## Results

### Characteristics of the included studies

The initial literature search identified 4762 studies of interest. After removing duplicates and screening all studies that applied the inclusion criteria, 119 studies were included in the quantitative meta‐analysis, for a total of 140 classification models, comprising 12,723 cases of SSD and 13,196 HCs (Fig. 1). Between Cheng et al.^90^ and Lei et al.^123^ there was an overlap of <20% of patients. This was considered a minor overlap, and both samples were included in the analysis. In Castro et al.^136^ and Antonucci et al.^145^, more than one model achieved the same best classification accuracy; thus, we averaged SE and SP across models accordingly. Among the included studies, 35 (for a total of 43 classification models) used sMRI,^51^, ^52^, ^53^, ^54^, ^55^, ^56^, ^57^, ^58^, ^59^, ^60^, ^61^, ^62^, ^63^, ^64^, ^65^, ^66^, ^67^, ^68^, ^69^, ^70^, ^71^, ^72^, ^73^, ^74^, ^75^, ^76^, ^77^, ^78^, ^79^, ^80^, ^81^, ^82^, ^83^, ^84^, ^85^ 48 (for a total of 55 classification models) used rs‐fMRI,^86^, ^87^, ^88^, ^89^, ^90^, ^91^, ^92^, ^93^, ^94^, ^95^, ^96^, ^97^, ^98^, ^99^, ^100^, ^101^, ^102^, ^103^, ^104^, ^105^, ^106^, ^107^, ^108^, ^109^, ^110^, ^111^, ^112^, ^113^, ^114^, ^115^, ^116^, ^117^, ^118^, ^119^, ^120^, ^121^, ^122^, ^123^, ^124^, ^125^, ^126^, ^127^, ^128^, ^129^, ^130^, ^131^, ^132^, ^133^ 17 (for a total of 18 classification models) used task‐fMRI,^59^, ^134^, ^135^, ^136^, ^137^, ^138^, ^139^, ^140^, ^141^, ^142^, ^143^, ^144^, ^145^, ^146^, ^147^, ^148^, ^149^ 12 (for a total of 13 classification models) used DTI,^59^, ^78^, ^150^, ^151^, ^152^, ^153^, ^154^, ^155^, ^156^, ^157^, ^158^, ^159^ and 10 (for a total of 11 classification models) used a combination of them (multimodal MRI),^160^, ^161^, ^162^, ^163^, ^164^, ^165^, ^166^, ^167^, ^168^, ^169^ to build predictive models (Table S1–S10).

### Meta‐analytic results

**Fig. 2:**
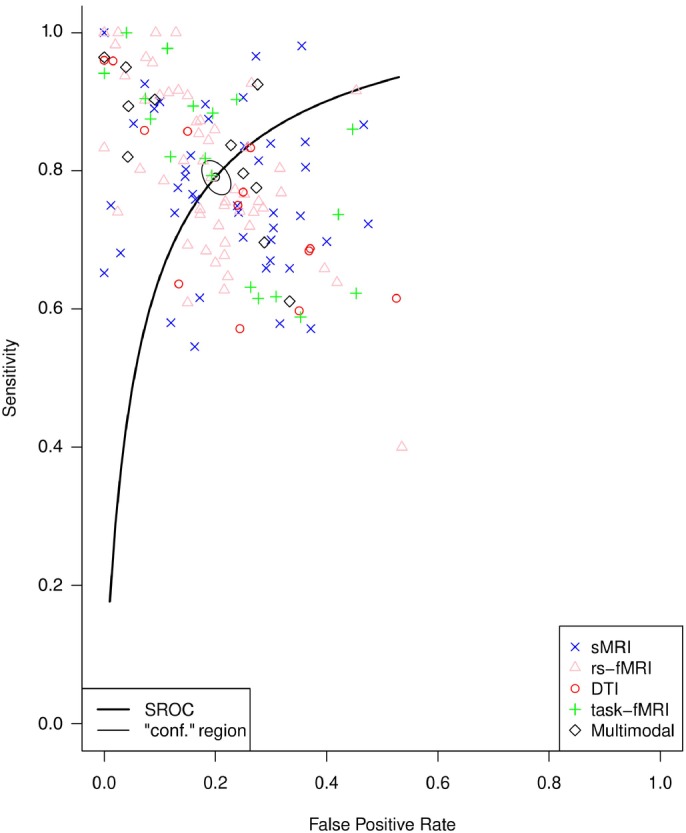
Summary receiver operating characteristic (SROC) curve of the Reitsma model with the summary sensitivity and false‐positive rate. DTI, diffusion tensor imaging; rs‐fMRI, resting‐state functional magnetic resonance imaging; sMRI, structural magnetic resonance imaging; task‐fMRI, task‐based functional magnetic resonance imaging.

Across all studies, the bivariate diagnostic random‐effects model estimated an SE of 79.1% (95% CI, 77.1%–81.0%) and an SP of 80.0% (95% CI, 77.8%–82.0%). A summary receiver operating characteristic (SROC) curve of the included studies along with the estimated summary is presented in Fig. 2, while the forest plots are reported in Figs S1 and S2. Although visual inspection of the funnel plot showed evidence of publication bias (Fig. S19), when regressing the inverted square root of ESS for logDOR, only a small part of the total heterogeneity between studies was explained (*R* ^2^ = 0.066, *P* = 0.002). When analyses were conducted by dividing the studies based on their data modality, SE and SP were as follows: 75.8% (95% CI, 66.6%–83.1%) and 80.5% (95% CI, 69.4%–88.2%) for DTI, 77.1% (95% CI, 73.5%–80.4%) and 77.5% (95% CI, 73.8%–80.9%) for sMRI, 79.7% (95% CI, 76.5%–82.5%) and 81.7% (95% CI, 78.4%–84.6%) for rs‐fMRI, 81.4% (95% CI, 74.3%–87.0%) and 77.8% (95% CI, 70.7%–83.7%) for task‐fMRI, and 83.7% (95% CI, 76.9%–88.8%) and 84.9% (95% CI, 75.6%–91.1%) for multimodal studies, respectively (Fig. S3). When stratifying for multimodality, we found that, SE and SP were 78.6% (95% CI, 76.4%–80.6%) and 79.5% (95% CI, 77.2%–81.6%) for single modality studies and 83.7% (95% CI, 76.9%–88.8%) and 84.9% (95% CI, 75.6%–91.1%) for multimodal neuroimaging studies, respectively (Fig. S4). Finally, we conducted two separate meta‐analyses for the stage of the disease: one in studies of patients with SSD in the first episode only and one in studies that included patients with chronic SSD only; SE and SP were 78.3% (95% CI, 73.6%–82.5%) and 78.2% (95% CI, 74.0%–81.9%) for studies with first episode and 79.1% (95% CI, 71.6%–85.1%) and 85.0% (95% CI, 76.8%–90.6%) for studies with chronic SSD, respectively (Fig. S5).

### Moderator analysis

We did not find any significant effect of sex, years of education, sample size, duration of illness, dose of medication, diagnostic criteria (*DSM* or *ICD*), type of scan, data modality, site type (single/multisite), first‐episode status, and inpatient status at the time of scan on SE or SP (all *P* > 0.05). The PANSS score, GAF score, age, age of onset, duration of untreated psychosis (DUP), classification algorithm, implementation of the DL methods, type of feature selection methods, and type of validation (internal vs external) showed significant effects. All meta‐regressions results are reported in the Table S11.

#### Effect of PANSS scores

We found a significant effect of the PANSS scores on performance accuracy. In particular, higher values of PANSS positive scores were correlated with higher SE (z = 2.815, *P* = 0.005) and SP (z = 4.025, *P* < 0.001) (Fig. S6), and the same was observed for PANSS negative scores on SP (z = 2.386, *P* = 0.017) (Fig. S7). In addition, we found a significant positive correlation between the PANSS positive‐to‐negative ratio and SE (z = 2.177, *P* = 0.030) and SP (z = 2.646, *P* = 0.008) (Fig. S8).

#### Effect of GAF scores

The GAF score showed a significant negative effect on SE (z = −2.563, *P* = 0.010) (Fig. S12).

#### Effect of age

The age of the patients was significantly correlated with SP (z = 2.737, *P* = 0.006); this correlation remained significant even when considering the age of the total sample (both SSD and HCs) (z = 2.063, *P* = 0.039) (Fig. S9).

#### Effect of age of onset

The age of disease onset was associated with SP (z = 2.151, *P* = 0.031) (Fig. S10).

#### Effect of the DUP

The DUP was significantly associated with SE (z = 2.030, *P* = 0.042) (Fig. S11).

#### Effect of feature selection methods

Filter methods were associated with better SE (z = 3.442, *P* = 0.001) and SP (z = 2.046, *P* = 0.041), while both performance metrics were significantly lower in studies without the feature selection procedure (z = −3.262, *P* = 0.001 and z = −2.455, *P* = 0.014, respectively). The summarized SE and SP were 86.4% (95% CI, 82.4%–89.6%) and 84.9% (95% CI, 80.3%–88.6%) for studies that used filter methods, and 75.3% (95% CI, 72.2%–78.2%) and 76.4% (95% CI, 72.9%–79.9%) for studies that did not employ any feature selection method, respectively. The comparison between the SROC curves is reported in Figs S17 and S18.

#### Effect of DL methods

The implementation of DL methods was significantly correlated with an overall improvement in classification performance for both SE (z = 2.371, *P* = 0.018) and SP (z = 2.519, *P* = 0.012). The summarized SE and SP were 85.4% (95% CI, 79.9%–89.6%) and 87.1% (95% CI, 79.8%–92.1%) for studies that implemented DL methods, and 78.1% (95% CI, 75.9%–80.2%) and 78.5% (95% CI, 76.3%–80.6%) for studies that did not, respectively. The comparison between the SROC curves is reported in Fig. S14.

#### Effect of algorithms

We found a significant effect of the type of algorithm used for the classification task. In particular, we conducted a meta‐regression on seven types of algorithms that were used in five or more studies (convolutional neural network [CNN], deep neural network [DNN], support vector machine [SVM], gradient boosting [G BOOST], decision tree [DT], linear discriminant analysis [LDA], and logistic regression [LR]). Liang et al.^109^, ^164^ used a Gradient Boosting Decision Tree; this algorithm was considered for meta‐regression both as G BOOST and decision tree algorithm, due to shared similarity to them; Wiem and Ali^118^ used deep CNN; for the same reason, this was considered as a regressor both for DNN and for CNN. The results showed that the use of CNN led to a better SE performance (z = 2.162, *P* = 0.031), while the use of LR led to a poorer performance in SE (z = −2.879, *P* = 0.004). The summarized SE and SP were 89.2% (95% CI, 79.4%–94.6%) and 88.1% (95% CI, 74.8%–94.8%) for CNN‐using studies, and 68.3% (95% CI, 59.1%–76.3%) and 72.9% (95% CI, 62.4%–81.4%) for studies that used LR for classification, respectively. The comparison between SROC curves is reported in Figs S15 and S16.

#### Effect of validation (internal vs external)

We found a significant difference in classification accuracy between internal and external validation using an independent data set. External validation was correlated with a decrease of SE (z = −2.710, *P* = 0.007), while no effect was found on SP. The summarized SE and SP were 69.2% (95% CI, 60.8%–76.5%) and 74.2% (95% CI, 64.1%–82.3%) for externally validated studies, and 79.9% (95% CI, 77.8%–81.8%) and 80.5% (95% CI, 78.3%–82.6%) for internally validated studies, respectively. The comparison between the SROC curves is reported in Fig. S13.

## Discussion

This meta‐analysis estimated the accuracy of ML methods applied to sMRI, DTI, rs‐fMRI, task‐fMRI, and multimodal MRI data to classify individuals with SSD. To our knowledge, this study is the first to systematically demonstrate the contribution of demographic, clinical, and model setting–related factors to the performance of the models. This evidence could inform future studies that aim to translate models into clinical settings and explore the impact of clinical variables on brain phenotypes.

Previous meta‐analyses provide a strong case for the use of ML for classifying patients who have SSD with neuroimaging measures.^30^, ^170^, ^171^ Their approaches show a partial overlap with our study for several reasons, including the type of MRI modalities, the heterogeneity of diagnosis within the SSD domain, the robustness of the ML approaches, and methodological differences in the handling of overlapping samples that are frequent in this literature. In particular, our study uniquely investigated all MRI methods (sMRI, rs‐fMRI, task‐fMRI, DTI) both individually and in combination, treating each modality as a moderator factor. Overall, our approach aligns with that used by Kambeitz et al.^30^, which we intended to update given the rapid growth of this field in the past decade. Our broader data set (comprising 119 studies and 140 classification models, with a total of 12,723 patients and 13,196 HCs, compared with 38 studies and 40 classification models, with 1602 patients and 1637 HCs) allowed us to explore additional factors (such as DUP, validation techniques, feature selection methods, and implementation of DL methods), yielding more precise insights relative to this work. Furthermore, the study by Kambeitz and colleagues also included positron emission tomography studies, which we excluded to reduce heterogeneity. Although we used similar methods to handle studies with overlapping samples, the inclusion of larger and more recent studies resulted in an overlap of only 11 of 119 studies included in our analysis.

Our study also differs from that of two more recent studies by Porter et al.^170^ (see below) and Wang et al.^171^. Specifically, Wang and colleagues, while restricting their analysis to the task‐fMRI modality, also included the at‐risk mental state for the psychosis category, which we excluded to ensure a more homogeneous and clinically relevant sample, thus improving the reliability of our findings. Furthermore, they considered multiclass classification studies, which we excluded to ensure robust comparability based solely on binary classification tasks. A crucial difference is the lack of addressing overlapping samples, which may have led to an overestimation of the performance. Overall, our study with the inclusion of brain MRI studies and a more focused diagnostic scope provides a more detailed understanding of how different MRI techniques, individually and collectively, contribute to the classification of SSD.

Although drawn from a larger sample of studies, our results are consistent with a recent meta‐analysis, which evaluated a subset of studies performing whole‐brain analysis on structural and resting‐state functional connectivity,^170^ and reported a relatively greater overall SE and SP, reaching ∼80%. Furthermore, moderator analysis showed a significant effect on the classification accuracy of PANSS scores, GAF scores, age, age of onset, DUP, classification algorithm, implementation of DL methods, type of algorithms employed, feature selection methods, and validation. Compared with the previous meta‐analysis, which did not find any significant effect of demographics and model characteristics on classification performance (except for a slight advantage for resting‐state functional connectivity relative to structural‐based modality on classification performance in specific subgroups of studies), the differences in the results observed in our study could be attributed to different methodological approaches. First, our analysis included global and regional neuroimaging studies, not only those focused on specific intrinsic brain networks and the whole brain. The rationale behind this decision was to obtain a comprehensive overview of classification performance using any features for each methodology. Indeed, in a more agnostic approach, we aimed to account for the potential confounding factor represented by the feature selection strategy adopted in different studies. This approach allowed us to mitigate feature selection biases associated with both a priori or ‘hypothesis‐driven’ selection of specific regions of interest (ROIs) or brain networks, as well as fully data‐driven approaches such as filters and wrappers, which may be susceptible to methodological limitations such as training sample overfitting or lack of generalizability. Although whole‐brain studies can be based both on voxel and ROI, in this context, we consider ROI‐based features as an *a priori* method of feature reduction. Given our goal of evaluating the classification power of each technique, we treated all of these methodologies equivalently. Notably, we regarded the selection of specific ROIs (instead of the parceled whole brain) as a form of a priori feature selection method.^22^ This aspect is particularly relevant, as we explored the impact of these diverse feature selection methods (including the absence of feature selection) through moderator analyses. Second, we did not conduct separate analyses for internal and external validation but opted for a unified analysis for consistency. This choice stems from the conceptual understanding that each form of model validation (either internal or external) is an evaluation of the generalizability of a statistical model.^172^ Thus, the moderating effect of these two conditions (internal vs external validation), similarly to other covariates, can be studied through moderator analysis. Third, when studies used overlapping samples, we prioritized those with larger sample sizes to bolster the representativeness and robustness of our analysis. This decision was rooted in the acknowledgment that ML classification benefits significantly from working with larger cohorts, and larger sample sizes can provide a more comprehensive and diverse data set, thus improving the reliability and generalizability of the findings.^173^ In addition, when studies developed multiple models within the same data modality using the same data set (with the same sample size), we selected the model with the best performance rather than averaging the performance. This decision aimed to standardize the best‐performing models for each data set before assessing the moderating effect of the covariates. Averaging various models in this context could obscure the effect of covariates, especially considering that some studies often employed several methods in a “trial‐and‐error” approach to maximize the prediction from their data. These studies may selectively present only the most favorable results or test alternative models with different algorithms, maintaining the same parameter settings that proved effective for the best‐performing model but might be incorrect for others,^174^ thus inflating results or masking the moderation effects of other variables. Last, because of the different inclusion criteria, only 34 of the 119 articles included in our final sample overlapped with those used in the previous meta‐analysis. Taken together, these differences could reasonably explain the conflicting results obtained. In conclusion, through broader inclusion criteria and a specific focus on SSD, coupled with updated analysis methodologies, our study offers a comprehensive and current understanding of this field. By addressing the limitations observed in earlier studies, we have uncovered new insights into the impact of various factors on ML model performance, contributing to the advancement of research in this critical area. In the following sections, we will discuss the performance of ML models and the effect of moderators.

### Performance of ML algorithms

Overall, patients with SSD were differentiated from HCs with an SE of 79.1% and an SP of 80.0%. These results align with previous meta‐analytic evidence showing that brain functional and structural alterations differentiated patients with SSD from HCs with an SE of 80.3% and an SP of 80.3%,^30^ demonstrating that neuroimaging‐based classification algorithms can accurately detect the disease condition.

### Role of clinical symptoms

The moderator analysis showed a positive correlation between PANSS positive scores on SE and SP and between PANSS negative scores on SP; thus, the classification models presented better classification performances when patients had more pronounced positive (e.g. hallucinations and delusions) or negative (e.g. social withdrawal and lack of affect) symptoms. More precisely, the positive symptoms, inherently associated with increased SE, demonstrated a correlation with a brain phenotype that mitigates the potential misclassification of individuals with SSD as HCs. Last, when considering the PANSS positive‐to‐negative ratio, a complementary measure indicating the relative symptomatology of each patient (e.g. the relative dominance of positive or negative symptoms rather than their absolute scores), we found that patients with a more positive symptom profile were better classified in terms of SE and SP. This result could be driven by the ability of functional imaging measures to capture altered responses associated with behavior and thought processes that are present in positive symptoms.^175^, ^176^ Furthermore, recent observations have highlighted the neurobiological differences between the predominant positive and negative schizophrenia subtypes, allowing the prediction of clinical phenotypes by rs‐fMRI. This is mainly attributed to distinct functional connectivity profiles in critical regions such as the ventromedial frontal cortex, the temporoparietal junction, and the precuneus.^177^ In addition, increased SE was associated with lower GAF scores, indicating a lower level of functioning, which could, at least partially, be an effect of cumulative neurobiological changes^178^ occurring throughout the course of schizophrenia and not only a maladaptive consequence of the illness. This is consistent with recent studies showing the role of functioning in the discrimination of schizophrenia from other patient populations^179^ and in the clustering of distinctive subgroups of individuals with psychosis.^180^

### Role of age, age of onset, and DUP

In our study, older age was associated with better SP. This finding might result from the accelerated brain aging in patients with SSD, which leads to more pronounced brain changes in older individuals with SSD compared to age‐matched HCs,^181^, ^182^ emerging as an important neuroanatomical signature for schizophrenia.^183^, ^184^ In addition, this result may be attributable to factors related to the chronicity of the illness, including smoking, substance use, and medication exposure,^185^, ^186^, ^187^ but not the current antipsychotic dose. Furthermore, we observed that the age of onset was positively correlated with increased SP. Importantly, there is ample evidence that SSDs are characterized by an acceleration of normal neurodevelopmental processes, which leads to widespread disruptions in gray and white matter and associated relevant functions.^188^ This result seems to align with the moderator effect of age previously discussed, possibly caused by various neurobiological alterations reflecting accelerated brain aging rather than neurodegenerative processes.^189^ Accelerated brain aging, in fact, was also described in the at‐risk stage, before the onset of the disease, and it appears not to depend on the age of onset itself.^184^ Lastly, DUP, the time between the development of the first psychotic symptoms and treatment,^190^ but not the duration of the illness, was associated with better SE. This result is not surprising, as DUP has been associated with changes in brain structure and function^191^, ^192^, ^193^ and poor clinical and social outcomes.^194^ Notably, this converges with the observed association between SE and decreased functioning assessed with GAF scores. Overall, our results suggest that the classification of patients with SSD (i.e. distinguishing SSD from HCs) using ML shows greater global accuracy in case of more pronounced positive symptoms, and greater SE when poor outcomes and functioning are present. Conversely, accelerated brain aging and negative symptoms enhance the ability of ML to classify HCs (i.e. distinguishing HCs from those with SSD). This could be explained by considering two distinct phenotypes: one characterized by a greater negative dimension where the effects of aging prevail over the neurodegenerative effects of the disease, which the models recognize as different from HCs but fail to characterize as SSD, and another one, characterized by predominantly positive symptoms and poorer functioning, which the models recognize more accurately as SSD. It is noteworthy that, consistent with our work, in a recent study on brain subtyping based on sMRI data using data‐driven clustering, Dwyer and colleagues^195^ found two distinct schizophrenia subgroups, with one characterized by older age, later age of onset, and relatively more severe negative symptoms. Classification accuracy between patients and HCs was improved by this stratification.^195^ In another study on rs‐fMRI data, ML applied to multisite data successfully identified a factorized structure based on symptomatic dimensions and predicted subtype membership from functional connectivity data.^169^ Taken together, these results highlight the potential of ML in unraveling the clinical and neurobiological heterogeneity of SSD. They also underscore its ability to uncover hidden patterns linking functional and neuroanatomical structures with clinical and neurocognitive profiles that appear to be reproducible and hold particular significance when supervised models classify patients with SSD and HCs. Such insights could significantly improve our understanding of the disease and, when recognized and incorporated into the model‐building process, could improve the overall performance, crucially for implementation in real‐life clinical settings.

### Role of ML and DL method

The outcomes of our meta‐regression analysis suggest that three methodological aspects of the classification model significantly impact its performance.

*Choice of an algorithm within the model*:

Specifically, the application of DL and CNN (particularly in terms of SE), appears to enhance performance in the classification task. This finding supports the superiority of DL over traditional ML when dealing with high dimensionality, such as in brain MRI data.^196^, ^197^

*Feature selection method*:^198^

Filter methods are significantly correlated with increased performance compared with embedded and multivariate techniques. This underscores the role of specific brain features, not necessarily spatially discriminated, in determining distinctive brain phenotypes. Conversely, it highlights the potential confounding role of other brain features that could negatively impact discrimination. This observation aligns with algorithm choice, as DL automatically learns to extract optimal features, overcoming challenges such as the *curse of dimensionality* and *small‐n‐large‐p* effects on performance. The superiority of filter methods can be attributed to their reduced risk of overfitting compared with methods based on hard feature selection, such as wrappers.^199^ It is crucial to emphasize our choice to include only studies employing a validation approach, either internal or external, thereby excluding studies presenting models that could have overfitted the training set.

*External validation*:^200^

The use of this approach is associated with reduced model SE, leading to a higher rate of patients with SSD misclassified as HCs. Consistent with the ML literature, these results emphasize the need to validate models trained in a specific cohort in separate samples, potentially selected from different clinical samples. This evidence suggests a possible overestimation of classification performance between studies, which indicates limited generalizability. Limitations such as selection bias in specific clinical settings, site effects related to data collection (e.g. type of MRI machine used and processing pipelines), biological variance across different ethnicities, and environments are among the factors impacting the replicability of classification algorithms across diverse populations. External validation is crucial to address these limitations and facilitate the development of clinically applicable models for practice. Not surprisingly, external validation studies showed a significantly larger confidence interval than internal validation studies. This could be attributable to a smaller number of studies and the high variability of the generalizability of different techniques and algorithm types.

### Limitations

This meta‐analysis has some limitations. First, we selected studies based on a categorical diagnosis, possibly overlooking the complexity of psychosis and thus missing the brain signatures of specific symptomatic dimensions of psychosis as a syndrome. However, most ML studies in schizophrenia have been performed using a dichotomous classification (patients vs. controls).^201^ Second, we conducted a meta‐analysis of the accuracy of ML, treating it as a specific measure while grouping different methods with potentially different population means of SE and SP. This approach may have masked variations in performance among slightly different ML methods. However, such an approach that allowed us to perform a more reliable estimation of accuracy could have resulted in a reduction, if any, and not an increase in the overall performance of ML methods. Third, there was considerable heterogeneity in the methodologies employed in the meta‐analyses, including variations in imaging techniques, preprocessing pipelines, and diagnostic criteria. The lack of a gold standard in the clinical and neuroimaging literature in the field makes it challenging to synthesize results and draw definitive conclusions. Fourth, the prevalence of studies conducted in specific populations or settings could have increased the reliability at the price of reduced generalizability of the results. Finally, current antipsychotic treatment could not represent a reliable measure of exposure to medication, and alternative parameters could more accurately reflect cumulative lifetime antipsychotic exposure to the brain. Unfortunately, lifetime exposure could be estimated only in longitudinal naturalistic studies of patients with first‐episode psychosis,^202^ while most classification studies in schizophrenia are cross‐sectional in nature.

Despite these limitations, our meta‐analysis provides valuable information on the current state of ML applications in psychosis research.

## Conclusions

In conclusion, our study emphasizes the effectiveness of multivariate pattern recognition methods in discerning dependable neuroimaging‐based biomarkers. Despite the various clinical manifestations within SSD, the distinctive functional and structural alterations in the brain successfully distinguish individuals with SSD from HCs, achieving an ∼80% SE and SP. Moreover, individual and methodological factors have been demonstrated to mediate the performance of the MRI‐based classification of SSD. In the future, these mediators should be rigorously addressed in ML models to ensure the performance and generalizability standards necessary for their translation into clinical practice.

## Disclosure statement

The authors have no conflicts of interest to declare.

## Author contributions

Fabio Di Camillo: Conceptualization, Literature review, Formal analysis, Writing – original draft. David Antonio Grimaldi: Formal analysis, Conceptualization, Writing – original draft. Giulia Cattarinussi: Literature review, Writing – original draft. Annabella Di Giorgio: Literature review, Writing – Review & Editing. Clara Locatelli: Literature review, Writing – Review & Editing. Adyasha Khuntia: Writing – Review & Editing. Paolo Enrico: Writing – Review & Editing. Paolo Brambilla: Writing – Review & Editing. Nikolaos Koutsouleris: Conceptualization, Writing – Review & Editing. Fabio Sambataro: Conceptualization, Formal analysis, Writing – original draft.

## Supporting information


**Data S1.** Supporting Information.
