# Supplementary material for: Magnetic resonance imaging–based machine learning classification of schizophrenia spectrum disorders: a meta‐analysis
Source: Psychiatry Clin Neurosci. 2024 Sep 18;78(12):732–43. doi: 10.1111/pcn.13736 (PMC11612547; doi:10.1111/pcn.13736)
Supplement: Supplementary file 1 — Data S1. Supporting Information. [file PCN-78-732-s001.docx]

*Title:* **MRI-based machine learning classification of schizophrenia spectrum disorders: a meta-analysis**

*Supplementary Material*

Contents:

Supplementary Tables S.1-S.11

Supplementary Figures S.1-S.20

**Tab. S1 – sMRI studies – Samples Characteristics.**

| **Author, Year** | **SCZ nr.** | **HC nr.** | **SCZ F nr. (%)** | **HC F nr. (%)** | **SCZ Age (SD)** | **HC Age (SD)** | **Education SCZ nr. (SD)** | **Education HC nr. (SD)** | **CPZ eq. (SD)** | **SCZ status** | **Inpatients/**  **Outpatients** | **Illness Duration mean (SD)** | **DUP mean (SD)** | **Age at Onset mean (SD)** | **PANSS Total mean (SD)** | **PANSS Positive mean (SD)** | **PANSS negative mean (SD)** | **GAF** | **Diagnostic Criteria** |
| --- | --- | --- | --- | --- | --- | --- | --- | --- | --- | --- | --- | --- | --- | --- | --- | --- | --- | --- | --- |
| Davatzikos 2005 | 69 | 79 | 23 (33.33) | 38  (48.10) | 29.90 (8.40) | 28.20 (7.50) | 13.50  (2.60) | 15.80  (2.20) | 37/69 taking AP | 32 FE, 37 Chronic | NA | 5.60  (6.30) | NA | NA | NA | NA | NA | NA | DSM-IV |
| Yushkevich 2005 | 46 | 46 | 16 (34.78) | 16  (34.78) | 31.05 (10.39) | 30.76 (10.45) | NA | NA | NA | NA | NA | NA | NA | NA | NA | NA | NA | NA | NA |
| Kawasaki 2007 | 30 | 30 | 0 (0) | 0 (0) | 24.70 (4.40) | 25.40 (4.40) | 13.30  (1.90) | 15.60  (1.90) | 435.00 (420.00) | NA | Inpatient and Outpatient | 4.00  (4.70) | NA | 21.10  (4.30) | NA | NA | NA | NA | DSM-IV, ICD-10 |
| Kawasaki 2007(a) | 16 | 16 | 0 (0) | 0 (0) | 28.60 (5.20) | 24.00 (5.10) | 14.10  (1.60) | 16.40  (1.60) | 605.00 (370.00) | NA | Inpatient and Outpatient | 5.10  (4.80) | NA | 23.10  (4.70) | NA | NA | NA | NA | DSM-IV, ICD-10 |
| Santos 2010 | 43 | 25 | NA | NA | NA | NA | NA | NA | NA | NA | Outpatient | NA | NA | NA | NA | NA | NA | NA | DSM-IV |
| Takayanagi 2011 | 29 | 22 | 0 (0) | 0 (0) | 27.83 (9.07) | 30.14 (8.21) | NA | NA | 1009.30 (802.94) | FE | Inpatient | 0.89  (1.43) | NA | NA | NA | NA | NA | NA | ICD-10 |
| Takayanagi 2011(a) | 23 | 18 | 23  (100) | 18  (100) | 28.15 (10.37) | 27.75 (6.12) | NA | NA | 814.06 (523.05) | FE | Inpatient | 1.12  (1.96) | NA | NA | NA | NA | NA | NA | ICD-10 |
| Wang 2011 | 32 | 32 | 7  (21.88) | 11  (34.38) | 24  (5.66) | 22.52 (4.20) | 11.15  (2.50) | 13.32  (3.50) | 27/32 taking AP | NA | Inpatient and Outpatient | 2.27  (1.39) | NA | NA | 80.06  (16.55) | NA | NA | NA | DSM-IV |
| Castellani 2012 | 54 | 54 | 35 (64.81) | 29  (53.70) | 37.96 (10.90) | 39.19 (10.05) | NA | NA | 241.90 (176.29) | NA | NA | 12.53  (10.04) | 1.76  (3.95) | NA | NA | NA | NA | NA | DSM-IV |
| Ota 2012 | 23 | 23 | 23  (100) | 23  (100) | 42.40 (13.20) | 46.40 (9.70) | 13.40  (2.50) | 13.90  (2.60) | 594.90  (534.90) | NA | NA | NA | NA | 23.70  (9.50) | NA | NA | NA | NA | DSM-IV |
| Pettersson-Yeo 2013 | 19 | 19 | 7  (36.84) | 7  (36.84) | 24.37 (4.71) | 24.89 (4.41) | NA | NA | 204.08 (116.35) | FE | NA | < 2 | NA | NA | 54.37  (15.13) | 12.58  (3.96) | 13.79  (5.26) | NA | DSM-IV |
| Gould 2014 | 200 | 134 | 56 (28.00) | 66  (49.25) | 38.05 (14.37) | 38.66 (12.70) | 13.65  (3.61) | 13.65  (3.61) | 178/200 taking AP | NA | Inpatient and Outpatient | NA | NA | 23.70  (9.05) | NA | NA | NA | 54.86 (18.03) | ICD-10 |
| Radulescu 2014 | 27 | 24 | 2  (7.41) | 1  (4.17) | 31.11 (9.33) | 32.17 (8.90) | NA | NA | 2 Drug naïve, 21/25 taking atypical AP | NA | NA | 6.07  (6.03) | NA | NA | NA | NA | NA | NA | DSM-IV-TR |
| Guo 2016 | 98 | 83 | 18 (18.37) | 13  (15.66) | 30.38 (8.68) | 29.48 (8.72) | NA | NA | 389.90 (501.70) | Various stages of illness | NA | 5.54  (6.21) | NA | NA | NA | NA | NA | NA | DSM-IV |
| Ou 2016 | 45 | 45 | 18 (40.00) | 20  (44.44) | 23.24 (4.63) | 23.64 (2.74) | 11.65  (2.57) | 12.13  (2.42) | NA | FE | Outpatient | 1.07 (0.53) | NA | NA | 90.10  (10.94) | 22.23  (5.56) | 22.10  (6.43) | NA | DSM-IV-TR |
| Yun 2016 | 27 | 96 | 18 (66.67) | 39  (40.63) | 22.48 (4.95) | 24.07 (4.84) | 12.85  (1.97) | 14.36  (1.69) | 327.47 (522.24) | FE | NA | 0.60  (0.52) | 0.51  (0.55) | 22.00  (4.97) | 73.00  (13.90) | 22.81  (5.42) | 18.81  (6.02) | NA | DSM-IV-TR |
| Dluhos 2017 | 258 | 222 | 61 (23.64) | 64  (28.83) | 26.02 (11.68) | 26.52 (11.87) | NA | NA | NA | FE | Inpatient and Outpatient | 1.31  (2.55) | NA | NA | NA | NA | NA | NA | ICD-10, DSM-IV |
| Liu 2017 | 38 | 38 | 13 (34.21) | 13  (34.21) | 25.00 (4.95) | 24.76 (4.56) | 10.39  (2.86) | 11.05  (2.91) | NA | FE | Outpatient | NA | NA | NA | NA | NA | NA | NA | NA |
| Salvador 2017 | 128 | 127 | 54 (42.19) | 54  (42.52) | 41.50 (10.30) | 39.80 (10.30) | NA | NA | 824.00 (642.80) | NA | Inpatient and Outpatient | 18.40  (11.00) | NA | NA | 72.60  (17.50) | 16.90  (5.70) | 21.40  (7.00) | NA | DSM-IV |
| Rozycki 2018 | 387 | 448 | 148 (38.24) | 215 (47.99) | 35.30 (11.00) | 33.90 (11.60) | NA | NA | 436.00  (NA) | NA | NA | 10.78  (NA) | NA | NA | NA | NA | NA | NA | NA |
| Schwarz 2019 | 375 | 368 | 120 (32.00) | 117 (31.79) | 34.41 (16.52) | 34.30 (19.0) | NA | NA | NA | NA | Inpatient and Outpatient | NA | NA | NA | NA | NA | NA | NA | DSM-IV, DSM-IV-TR |
| Winterburn 2019 | 88 | 103 | 30 (34.09) | 46  (44.66) | 36.60 (12.40) | 35.20 (12.40) | 13.30  (2.33) | 15.50  (1.91) | NA | NA | NA | 12.59  (12.27) | NA | 24.00  (6.78) | 53.78  (11.04) | 14.18  (5.98) | 14.27  (6.11) | NA | DSM-IV |
| Winterburn 2019(a) | 50 | 50 | 19 (38.00) | 18  (36.00) | 25.80 (7.35) | 23.80 (4.79) | 11.90  (3.26) | 15.60  (2.57) | 0 (0) | FE | Inpatient and Outpatient | 0.67  (1.07) | 0.67  (1.07) | 24.70  (7.51) | 97.60  (11.52) | 23.90  (4.97) | 24.30  (5.83) | NA | DSM-IV |
| Chang 2020 | 200 | 200 | NA | NA | NA | NA | NA | NA | 385.70 (329.60) | NA | NA | 15.50 (10.90) | NA | 28.20 (10.10) | 40.80  (11.60) | NA | NA | NA | DSM-IV |
| Chang 2020(a) | 88 | 44 | NA | NA | NA | NA | NA | NA | NA | NA | NA | 22.80  (11.30) | NA | NA | 72.30  (15.60) | NA | NA | NA | DSM-IV |
| Lei 2020 | 32 | 83 | 8  (25.00) | 45  (54.22) | 40.94 (10.90) | 28.09 (8.98) | 14.72  (4.41) | 17.73  (3.28) | 27/32 taking AP | Chronic | Inpatient and Outpatient | NA | NA | NA | NA | NA | NA | NA | DSM-IV |
| Oh 2020 | 30 | 30 | NA | NA | NA | NA | NA | NA | 30/30 taking AP | NA | NA | 4.89  (3.47) | 3.67  (3.81) | NA | 54.90  (28.40) | NA | NA | 62.80 (12.30) | NA |
| Vieira 2020 | 110 | 110 | 42 (38.18) | 42  (38.18) | 28.50 (8.60) | 29.70 (7.80) | NA | NA | NA | FE | Inpatient and Outpatient | 0.30  (0.70) | NA | NA | NA | NA | NA | NA | DSM-IV |
| Vieira 2020(a) | 140 | 70 | 50 (35.71) | 25  (35.71) | 28.30 (7.60) | 27.30 (7.50) | NA | NA | NA | FE | Inpatient and Outpatient | 0.30 (0.90) | NA | NA | NA | NA | NA | NA | DSM-IV |
| Yamamoto 2020 | 99 | 99 | 50 (50.51) | 47  (47.47) | 33.50 (8.520) | 31.85 (7.83) | 13.55  (3.42) | 15.28  (2.05) | 513.87 (582.21) | NA | NA | 10.10  (9.50) | NA | 23.31  (8.18) | 65.96  (31.72) | 14.71  (8.56) | 17.54  (10.42) | NA | DSM-IV |
| Li 2021 | 89 | 83 | 24 (26.97) | 26  (31.33) | 24.12 (6.95) | 21.45 (7.37) | 13.32  (NA) | 11.45  (NA) | 0 (0) | FE | NA | 1.21 (1.09) | 1.21 (1.09) | NA | >60 | NA | NA | NA | DSM-V |
| Lieslehto 2021 | 29 | 61 | 14 (48.28) | 23  (37.70) | 42.80 (0.57) | 43.00 (0.54) | NA | NA | 354.63 (367.68) | Chronic | Outpatients | 19.83 (4.58) | NA | 22.90 (4.50) | NA | 15.61 (5.83) | 22.36 (11.39) | NA | DSM-III-R, DSM-IV |
| Lieslehto 2021(a) | 44 | 43 | 14 (31.82) | 23  (53.49) | 32.45 (6.91) | 31.53 (8.43) | 12.79  (1.82) | 15.96  (2.55) | NA | NA | NA | 12.35  (7.26) | NA | 20.65  (4.61) | NA | NA | NA | NA | DSM-IV |
| Morgan 2021 | 65 | 59 | 22 (33.85) | 36  (61.02) | 27.90 (6.50) | 29.20 (10.30) | NA | NA | 63/65 taking AP | NA | Inpatient and Outpatient | NA | NA | NA | NA | 9.40  (3.80) | 10.30  (5.40) | NA | DSM-IV |
| Zhou 2021 | 49 | 34 | 25 (51.02) | 19  (55.88) | 16.02 (1.80) | 16.32 (2.99) | NA | NA | NA | FE and Chronic | NA | NA | NA | 15.39  (2.10) | NA | NA | NA | NA | DSM-IV |
| Chilla 2022 | 158 | 76 | NA | NA | NA | NA | NA | NA | 205.44 (184.50) | NA | NA | 6.50  (7.36) | NA | NA | NA | NA | NA | NA | DSM-IV |
| Cui 2022 | 662 | 613 | 300 (45.32) | 297 (48.45) | 27.50 (86.50) | 27.50 (6.10) | NA | NA | 411.00 (204.30) | FE/Relapse 269/393 | NA | 4.13  (4.30) | NA | 23.50  (6.10) | NA | 24.00  (4.20) | 20.50  (6.20) | 46.70 (13.80) | DSM-IV-TR |
| Korda 2022 | 77 | 44 | NA | NA | NA | NA | NA | NA | NA | FE | NA | NA | NA | NA | NA | NA | NA | NA | DSM-III-R |
| Xie 2022 | 514 | 653 | 160 (31.13) | 290 (44.41) | 34.72 (30.98) | 33.42 (31.46) | NA | NA | NA | NA | NA | NA | NA | NA | NA | NA | NA | NA | DSM-IV |
| Xie 2022(a) | 69 | 34 | 40 (57.97) | 23  (67.65) | 33.62 (5.57) | 32.62 (6.03) | NA | NA | NA | NA | NA | NA | NA | NA | NA | NA | NA | NA | DSM-IV |
| Knolee 2023 | 146 | 145 | 58 (39.73) | 59  (40.69) | 22.83 (4.07) | 23.67 (3.93) | NA | NA | 91/146 taking AP | FE/ROS | NA | NA | NA | NA | 46.10  (13.78) | NA | NA | NA | ICD-10 |
| Sun 2023 | 158 | 166 | 84 (53.16) | 79 (47.59) | 23.68 (7.42) | 23.62 (6.03) | 14.86 (3.41) | 15.05 (3.73) | NA | FE | NA | < 2 | NA | NA | 89.33  (14.16) | 18.73   (7.97) | 24.71  (6.36) | 30.10  (11.13) | DSM-IV |
| Sun 2023(a) | 77 | 58 | 41 (53.25) | 30  (51.72) | 24.37 (4.48) | 24.21 (5.65) | 14.32  (3.13) | 14.68  (2.72) | NA | FE | NA | < 2 | NA | NA | 85.68  (13.62) | 18.15  (7.34) | 23.24  (6.57) | 32.02  (12.58) | DSM-IV |

AP: antipsychotic, FE: first-episode, ROS: recent-onset schizophrenia.

**Tab. S2 - rs-fMRI studies – Samples Characteristics.**

| **Author, Year** | **SCZ nr.** | **HC nr.** | **SCZ F nr. (%)** | **HC F nr. (%)** | **SCZ Age (SD)** | **HC Age (SD)** | **Education SCZ nr. (SD)** | **Education HC nr. (SD)** | **CPZ eq. (SD)** | **SCZ status** | **Inpatients/**  **Outpatients** | **Illness Duration mean (SD)** | **DUP mean (SD)** | **Age at Onset mean (SD)** | **PANSS Total mean (SD)** | **PANSS Positive mean (SD)** | **PANSS negative mean (SD)** | **GAF** | **Diagnostic Criteria** |
| --- | --- | --- | --- | --- | --- | --- | --- | --- | --- | --- | --- | --- | --- | --- | --- | --- | --- | --- | --- |
| Shi 2007 | 48 | 35 | 22  (45.83) | 15  (42.86) | 23.50 (6.60) | 27.10 (6.20) | NA | NA | 467.40 (215.50) | NA | Inpatient | 2.29  (3.22) | NA | NA | NA | NA | NA | NA | DSM-IV |
| Su 2013 | 32 | 32 | 7  (21.88) | 9  (28.13) | 24.00 (5.66) | 25.01 (4.50) | 11.15  (2.50) | NA | 27/32 taking AP | NA | Outpatient and Inpatient | 2.27  (1.39) | NA | NA | 80.06  (16.55) | NA | NA | NA | DSM-IV |
| Anticevic 2014 | 90 | 90 | 24  (26.67) | 31  (34.44) | 32.93 (11.25) | 30.71 (11.99) | 13.18  (2.21) | 15.24  (2.22) | 229.00 (195.81) | NA | Outpatient | NA | NA | NA | 60.51  (14.25) | 15.80  (4.73) | 14.34  (5.53 | NA | DSM-IV |
| Anticevic  2014(a) | 23 | 23 | 5  (21.74) | 5  (21.74) | 36.39 (9.54) | 37.18 (7.59) | 13.04  (2.14) | 15.26  (2.12) | 584.63 (563.63) | NA | Outpatient | NA | NA | NA | NA | NA | NA | NA | DSM-IV |
| Zhu 2014 | 27 | 28 | 15  (55.56) | 16  (57.14) | 22.90 (3.20) | 22.30 (3.80) | 9.80  (5.00) | 10.10  (4.10) | 27/27 taking AP | NA | NA | NA | NA | NA | 64.60  (NA) | NA | NA | NA | DSM-IV |
| Cheng 2015 | 415 | 405 | 172 (41.45) | 178  (43.95) | 27.62 (9.88) | 29.68 (9.82) | NA | NA | NA | NA | NA | NA | NA | NA | 72.43  (6.92) | 18.64  (5.08) | 17.1  (6.25) | NA | DSM-IV |
| Cheng 2015(a) | 19 | 29 | 6 (31.58) | 15 (51.72) | 33.10 (10.90) | 28.10 (8.40) | NA | NA | NA | NA | NA | NA | NA | NA | NA | NA | NA | NA | DSM-IV |
| Chyzhyk 2015 | 40 | 28 | NA | NA | NA | NA | NA | NA | NA | NA | NA | NA | NA | NA | NA | NA | NA | NA | DSM-IV-TR |
| Mueller 2015 | 31 | 37 | 7  (22.58) | 6  (16.22) | 34.70 (11.30) | 34.60 (11.70) | NA | NA | 298.00 (335.00) | NA | Outpatient | 11.90  (10.20) | NA | NA | 50.00  (13.60) | 12.90  (5.50) | 12.60  (5.20) | NA | DSM-IV |
| Mikolas 2016 | 63 | 63 | 24  (38.10) | 24  (38.10) | 28.80 (6.20) | 28.10 (6.30) | NA | NA | 375.00 (289.50) | FE | Inpatient | 0.17  (NA) | NA | NA | 70.10  (17.70) | 16.90  (6.70) | 16.90  (6.40) | NA | ICD-10 |
| Peters 2016 | 18 | 18 | 9  (50.00) | 9  (50.00) | 35.33 (12.49) | 34.10 (13.4) | NA | NA | 466.72 (440.49) | NA | Inpatient | 7.15 (6.89) | NA | NA | 76.44  (18.45) | 18.06  (5.74) | 19.94  (8.11) | 41.50 (11.55) | DSM-IV |
| Guo 2017 | 28 | 40 | 10  (35.71) | 20  (50.00) | 22.93 (3.92) | 23.28 (2.60) | 10.54  (2.32) | 11.53  (1.81) | 0 (0) | FE | NA | 2.01  (0.55) | 2.01  (0.55) | NA | 88.11  (10.29) | 22.68  (5.64) | 21.18  (5.40) | NA | DSM-5 |
| Skåtun 2017 | 182 | 348 | 60  (32.97) | 143  (41.09) | 33.31 (8.24) | 34.15 (7.54) | NA | NA | NA | NA | Outpatient and Inpatient | 8.67 (5.02) | NA | NA | NA | NA | NA | NA | DSM-IV |
| Kottaram 2018 | 41 | 41 | 13  (31.71) | 17  (41.46) | 40.90 (10.00) | 38.30 (10.50) | 12.00  (0.55) | 16.40  (0.47) | 615.40  (55.84) | Chronic | NA | 17.90  (9.30) | NA | NA | 59.10  (13.10) | 15.60  (6.58) | 16.40  (5.18) | 45.90 (13.00) | DSM-IV |
| Liu 2018 | 48 | 31 | 27  (56.25) | 17  (54.84) | 15.79 (1.64) | 15.42 (1.52) | 8.88  (1.95) | 8.44  (1.56) | 0 (0) | FE (AOS) | Outpatient | 0.45  (0.51) | 0.45  (0.51) | NA | 75.10  (9.88) | 21.50  (5.01) | 17.92  (6.95) | NA | DSM-IV-TR |
| Moghimi 2018 | 82 | 88 | 25  (30.49) | 27  (30.68) | 32.87 (12.92) | 33.31 (13.76) | NA | NA | 72/82 taking AP | FE and Chronic | NA | NA | NA | NA | NA | NA | NA | NA | NA |
| Ji 2019 | 66 | 85 | 39  (59.09) | 41  (48.24) | 33.40 (7.20) | 33.30 (9.70) | 10.20  (3.10) | 10.80  (3.90) | NA | NA | NA | 7.20  (6.40) | NA | NA | NA | NA | NA | NA | DSM-IV |
| Ji 2019(a) | 56 | 38 | 28  (50.00) | 24  (63.16) | 24.90 (6.20) | 27.30 (7.10) | 11.90  (2.80) | 11.60  (2.20) | NA | NA | NA | 4.40  (3.60) | NA | NA | 45.80  (8.80) | 11.10  (3.40) | 10.10  (3.30) | NA | DSM‐IV |
| Kalmady 2019 | 81 | 93 | 28  (34.57) | 33  (35.48) | 30.72 (6.16) | 29.41 (5.71) | NA | NA | 0 (0) | NA | NA | 3.08  (4.26) | 3.08  (4.26) | 26.60  (6.17) | NA | NA | NA | NA | DSM-IV |
| Qiu 2019 | 42 | 40 | 7  (16.67) | 12  (30.00) | 41.05 (14.16) | 36.25 (11.4) | NA | NA | NA | NA | NA | NA | NA | NA | NA | NA | NA | NA | NA |
| Shaoqiang 2019 | 41 | 34 | 15  (36.59) | 13  (38.24) | 24.98 (4.79) | 25.12 (4.58) | 10.37  (2.80) | 11.12  (2.80) | 0 (0) | FE | NA | 0.69  (0.22) | 0.69  (0.22) | NA | 92.39  (10.92) | 25.78  (3.60) | 18.32  (5.18) | NA | DSM-IV-TR |
| Zhu 2019 | 76 | 71 | 36  (47.37) | 35  (49.30) | 23.30 (6.00) | 21.37 (2.83) | 11.62  (2.70) | 15.00  (2.03) | 0 (0) | FE | NA | 0.91  (0.80) | 0.91 (0.80) | NA | 84.86  (20.69) | 19.73  (6.78) | 20.65  (8.59) | NA | DSM-IV |
| Cai 2020 | 51 | 51 | 30  (58.82) | 33  (64.71) | 43.22 (10.89) | 42.04 (12.17) | 12.07  (2.95) | 12.80  (3.73) | 236.56 (172.21) | NA | NA | 16.66  (8.07) | NA | 25.41  (9.19) | 51.69  (14.77) | 11.63  (4.86) | 13.39  (5.78) | NA | DSM-IV |
| Cai(a) 2020 | 34 | 27 | 6  (17.65) | 15  (55.56) | 36.5  (7.14) | 27.37 (7.34) | 12.18  (2.21) | 13.93  (2.81) | 287.05 (193.83) | NA | NA | 10.54  (7.87) | NA | 24.17  (6.64) | 67.72  (12.38) | 10.00  (3.87) | 23.16  (3.12) | NA | DSM-IV |
| Cao 2020 | 43 | 29 | 24  (55.81) | 16  (55.17) | 28.30 (9.90) | 27.70 (7.80) | NA | NA | 300.0 – 600.0 (NA) | FE | Inpatient | NA | NA | NA | NA | NA | NA | NA | DSM-IV |
| Ji 2020 | 205 | 240 | 71  (34.63) | 138  (57.50) | 35.35 (12.47) | 38.18 (12.70) | NA | NA | 181/201 taking AP | NA | NA | NA | NA | NA | NA | NA | NA | NA | DSM-IV-TR |
| Liang 2020 | 100 | 93 | 49  (49.00) | 58  (62.37) | 24.50 (6.49) | 24.68 (4.96) | 12.18  (2.95) | 15.33  (2.50) | 27/100 taking AP for <3days | FE | NA | 1.24  (1.67) | NA | NA | 79.62  (12.21) | 21.48  (7.56) | 20.60  (9.53) | NA | DSM-IV |
| Liang 2020(a) | 87 | 80 | 49  (56.32) | 41  (51.25) | 24.91 (7.96) | 26.13 (8.18) | 11.92  (3.31) | 12.89  (3.47) | 10/87 taking AP for <3days | FE | NA | 0.84  (1.67) | NA | NA | 90  (8.56) | 24.73  (6.69) | 18.96  (8.23) | NA | DSM-IV |
| Sartipi 2020 | 8 | 10 | NA | NA | NA | NA | NA | NA | NA | NA | NA | NA | NA | NA | NA | NA | NA | NA | NA |
| Wang 2020 | 23 | 23 | 13  (56.52) | 15  (65.22) | 32.10 (9.10) | 33.50 (7.70) | NA | NA | NA | NA | NA | NA | NA | NA | 98.00  (11.10) | NA | NA | NA | NA |
| Wang 2020(a) | 43 | 52 | 10  (23.26) | 20  (38.46) | 25.70 (7.80) | 25.90 (4.50) | 13.00  (3.40) | 14.00  (3.20) | 152.00  (146.00) | NA | NA | NA | NA | NA | 89.70  (12.30) | 24.30  (5.20) | 22.40  (6.50) | NA | DSM-IV |
| Yang 2020 | 72 | 54 | 24  (33.33) | 23  (42.59) | 23.4  (NA) | 23.5  (NA) | 11.6  (NA) | 14.8  (NA) | 0 (0) | FE | NA | NA | NA | NA | NA | NA | NA | NA | DSM-IV |
| Yoshihara 2020 | 68 | 102 | 35  (51.47) | 40  (39.22) | 38.40 (9.10) | 31.10 (9.60) | NA | NA | 568.90 (424.60) | Chronic | Outpatient and Inpatient | 12.80  (7.80) | NA | NA | 58.10  (17.40) | 13.80  (5.10) | 15.20  (5.60) | NA | DSM-IV |
| Yoshihara 2020(a) | 47 | 43 | 11  (23.40) | 30  (69.77) | 29.60 (7.40) | 28.40 (7.30) | NA | NA | 340.80 (208.10) | Chronic | NA | 5.90 (5.80) | NA | NA | 62.80 (10.50) | 15.90 (4.20) | 17.00 (5.20) | NA | DSM-IV |
| Yoshihara 2020(b) | 30 | 71 | 7  (23.33) | 35  (49.30) | 22.10 (4.60) | 24.60 (3.90) | NA | NA | 462.70 (346.50) | FE | NA | 1.40 (0.80) | NA | NA | NA | NA | NA | NA | DSM-IV |
| Lyu 2021 | 32 | 27 | 17  (53.13) | 17  (62.96) | 16.75 (1.22) | 16.40 (2.12) | 10.19  (1.66) | 10.11  (2.82) | 0 (0) | FE and AOS | NA | 0.77  (1.06) | 0.77  (1.06) | NA | 79.44  (15.95) | 22.75  (6.89) | 16.97  (7.48) | NA | DSM-IV |
| Serin 2021 | 12 | 15 | 2  (16.67) | 1  (6.67) | 32.80 (9.20) | 33.30 (9.20) | NA | NA | NA | NA | NA | NA | NA | NA | NA | NA | NA | NA | DSM-IV |
| Wang 2021 | 28 | 28 | 14  (50.00) | 17  (60.71) | NA | NA | NA | NA | NA | NA | NA | NA | NA | NA | NA | NA | NA | NA | NA |
| Wiem 2021 | 23 | 41 | NA | NA | NA | NA | NA | NA | NA | NA | NA | NA | NA | NA | NA | NA | NA | NA | NA |
| Feng 2022 | 45 | 45 | 28  (62.22) | 29  (64.44) | 23.00 (6.30) | 23.30 (2.30) | NA | NA | 0 (0) | FE | NA | NA | NA | NA | 66.50  (14.60) | 18.20  (3.80) | 17.20  (5.50) | NA | DSM-IV |
| Gao 2022 | 131 | 128 | 55  (41.98) | 53  (41.41) | 26.34 (10.39) | 30.33 (7.90) | 9.50  (2.64) | 9.30  (2.61) | NA | NA | Outpatient and Inpatient | 6.92  (4.86) | NA | 19.42  (12.21) | 89.63  (17.00) | NA | NA | NA | DSM-IV |
| Gao 2022(a) | 57 | 50 | 37  (64.91) | 27  (54.00) | 31.63 (11.43) | 28.38 (6.87) | 12.86  (3.42) | 15.64  (2.26) | 0 (0) | FE | NA | 2.52  (2.72) | 2.52  (2.72) | NA | 91.84  (14.16) | 26.39  (4.85) | 20.68  (6.89) | NA | ICD-10 |
| Lee 2022 | 220 | 220 | 100 (45.45) | 110  (50.00) | 31.70 (9.60) | 31.80 (9.70) | 13.20  (2.90) | 14.60  (2.70) | 207/220 taking AP | NA | Outpatient and Inpatient | 9.40  (8.10) | NA | 22.50  (6.80) | 66.30  (15.90) | 15.10  (4.90) | 17.40  (5.30) | NA | DSM-IV |
| Lei 2022 | 505 | 907 | 216 (42.77) | 482  (53.14) | 28.93 (10.3) | 29.33 (11.02) | NA | NA | NA | FE and Chronic | NA | NA | NA | NA | NA | NA | NA | NA | DSM-IV |
| Oh 2022 | 171 | 161 | 82  (47.95) | 87  (54.04) | 34.38 (10.61) | 33.73 (10.96) | 13.90  (2.44) | 15.26  (2.07) | 449.33 (351.495) | NA | NA | 6.48  (8.04) | NA | NA | 50.05  (23.26) | 13.69  (8.00) | 11.57  (6.55) | NA | DSM-IV-TR |
| Yang 2022 | 69 | 69 | 34  (49.28) | 34  (49.28) | 46.06 (10.96) | 45.84 (11.89) | NA | NA | NA | NA | NA | 19.84  (10.96) | NA | NA | 85.51  (9.50) | 20.06  (4.59) | 23.78  (3.84) | NA | DSM-IV-TR |
| Zhao 2022 | 558 | 542 | 266 (47.67) | 266  (49.08) | 27.60 (7.10) | 28.00 (7.20) | NA | NA | NA | NA | NA | NA | NA | NA | 83.60  (12.30) | 23.90  (4.20) | 20.10  (5.90) | NA | DSM-IV-TR |
| Ellis 2023 | 151 | 160 | NA | NA | NA | NA | NA | NA | NA | NA | NA | NA | NA | NA | NA | NA | NA | NA | DSM-IV |
| Hancock 2023 | 82 | 53 | 27  (32.93) | 19  (35.85) | 23.42 (3.57) | 24.85 (4.15) | NA | NA | NA | FE/ROS | NA | NA | NA | NA | 53.42  (10.02) | NA | NA | NA | DSM-5 |
| Huang 2023 | 178 | 180 | 88  (49.44) | 85  (47.22) | 27.80 (10.50) | 27.80 (12.50) | NA | NA | NA | NA | NA | NA | NA | NA | NA | NA | NA | NA | NA |
| Kang 2023 | 52 | 51 | 32  (61.54) | 29  (56.86) | 26.00 (6.88) | 26.00 (6.51) | 12.60  (2.99) | 13.00  (2.81) | 0 (0) | FE | Inpatient | NA | NA | NA | NA | NA | NA | NA | DSM-IV |
| Li 2023 | 94 | 101 | 41  (43.62) | 58  (57.43) | 33.23 (8.03) | 33.86 (10.77) | NA | NA | NA | NA | NA | NA | NA | NA | NA | NA | NA | NA | DSM‐IV‐TR |
| Ma 2023 | 44 | 40 | 12  (27.27) | 16  (40.00) | 20.48 (4.47) | 20.38 (2.65) | 11.68  (2.28) | 13.18  (2.46) | 0 (0) | FE | Outpatient | NA | NA | NA | 83.33  (16.69) | 20.83  (5.95) | 22.02  (7.27) | NA | DSM-IV |
| Ma 2023(a) | 11 | 10 | 3  (27.27) | 2  (20.00) | 20.82 (3.84) | 19.80 (3.05) | 10.64  (2.2) | 11.40  (2.22) | 0 (0) | FE | NA | NA | NA | NA | 82.46  (10.88) | 18.73  (4.15) | 21.27  (4.86) | NA | DSM-IV |
| Xing 2023 | 130 | 153 | NA | NA | NA | NA | NA | NA | NA | NA | NA | NA | NA | NA | NA | NA | NA | NA | NA |

AOS: adolescent-onset schizophrenia, AP: antipsychotic, FE: first-episode, ROS: recent-onset schizophrenia.

**Tab. S3 – DTI studies – Samples Characteristics.**

| **Author, Year** | **SCZ nr.** | **HC nr.** | **SCZ F nr. (%)** | **HC F nr. (%)** | **SCZ Age (SD)** | **HC Age (SD)** | **Education SCZ nr. (SD)** | **Education HC nr. (SD)** | **CPZ eq. (SD)** | **SCZ status** | **Inpatients/**  **Outpatients** | **Illness Duration mean (SD)** | **DUP mean (SD)** | **Age at Onset mean (SD)** | **PANSS Total mean (SD)** | **PANSS Positive mean (SD)** | **PANSS negative mean (SD)** | **GAF** | **Diagnostic Criteria** |
| --- | --- | --- | --- | --- | --- | --- | --- | --- | --- | --- | --- | --- | --- | --- | --- | --- | --- | --- | --- |
| Rathi 2010 | 21 | 20 | 4  (19.05) | 5  (25.00) | 21.21  (4.56) | 22.47  (3.48) | NA | NA | NA | FE | NA | NA | NA | NA | NA | NA | NA | NA | NA |
| Ardekani 2011 | 50 | 50 | 16  (32.00) | 16  (32.00) | 30.30 (10.50) | 31.20  (9.70) | NA | NA | NA | 33 FE, 17 Chronic | Inpatients and Outpatients | NA | NA | NA | NA | NA | NA | NA | DSM-IV |
| Petterson-Yeo 2013(a) | 19 | 19 | 7  (36.84) | 7  (36.84) | 24.37  (4.71) | 24.89  (4.41) | NA | NA | 204.08 (116.35) | FE | NA | < 2 | NA | NA | 54.37  (15.13) | 12.58  (3.96) | 13.79  (5.26) | NA | DSM-IV |
| Zheng 2013 | 49 | 63 | NA | NA | NA | NA | NA | NA | 0 (0) | FE | Outpatients | NA | NA | NA | NA | NA | NA | NA | DSM-IV-TR |
| Chen 2018 | 168 | 204 | 84  (50.00) | 104  (50.98) | 29.48 (11.63) | 29.48 (11.14) | 14.26  (4.75) | 15.79  (6.03) | 305.42 (367.47) | NA | NA | 8.62  (12.09) | NA | 23.70  (14.19) | 54.13  (25.33) | 12.93  (10.80) | 15.85  (11.75) | NA | DSM-IV |
| Mikolas 2018 | 77 | 77 | 34  (44.16) | 34  (44.16) | 28.32  (7.02) | 28.51  (7.03) | NA | NA | 337.00 (234.8) | FE | Inpatients | 0.25  (0.59) | NA | NA | 62.40  (16.70) | 13.90  (4.90) | 15.70  (6.10) | NA | ICD-10 |
| Deng 2019 | 65 | 60 | 33  (50.77) | 29  (48.33) | 27.60  (6.57) | 27.20  (6.38) | 12.54  (3.23) | 15.80  (2.81) | 338.90 (168.83) | FE | NA | 1.21  (1.28) | 0.87  (1.20) | NA | 45.20  (12.33) | 9.69  (3.43) | 11.55  (5.89) | NA | DSM-IV |
| Huang 2020 | 18 | 19 | 10  (55.56) | 13  (68.42) | 31.40  (9.50) | 33.90  (7.70) | NA | NA | NA | NA | NA | NA | NA | NA | 98.70  (11.60) | NA | NA | NA | NA |
| Lin 2021 | 99 | 69 | 60  (60.61) | 31  (44.93) | 29.84  (8.55) | 25.62  (5.95) | 12.68  (3.04) | 13.35  (4.26) | NA | FE and Chronic | NA | 5.58  (5.33) | NA | 24.31  (7.52) | 79.37  (7.33) | 26.12  (4.06) | 16.52  (3.69) | NA | DSM-IV-TR |
| Masouidi 2021 | 64 | 81 | 13  (20.31) | 21  (25.93) | 38.92  (NA) | 37.98  (NA) | NA | NA | NA | NA | NA | NA | NA | NA | NA | NA | NA | NA | DSM‐IV |
| Morgan 2021(a) | 33 | 82 | 9  (27.27) | 47  (57.32) | 42.20 (11.70) | 33.50 (12.69) | NA | NA | NA | NA | NA | NA | NA | NA | NA | 14.50  (4.20) | 12.50  (5.50) | NA | DSM‐IV |
| Morgan 2021(b) | 65 | 59 | 22  (33.85) | 36  (61.02) | 27.90  (6.50) | 29.20 (10.30) | NA | NA | NA | NA | Inpatient and Outpatient | NA | NA | NA | NA | 9.40  (3.80) | 10.30  (5.40) | NA | DSM‐IV |
| Wang 2022 | 140 | 205 | 95  (67.86) | 110  (53.66) | 34.22  (8.23) | 32.51  (8.39) | 10.69  (3.32) | 12.84  (2.83) | NA | 61 FE, 79 Chronic | NA | NA | NA | NA | 85.55  (18.55) | 23.14  (5.25) | 22.53  (7.48) | NA | DSM-IV-TR |

FE: first-episode.

**Tab. S4 - task-fMRI studies – Samples Characteristics.**

| **Author, Year** | **SCZ nr.** | **HC nr.** | **SCZ F nr. (%)** | **HC F nr. (%)** | **SCZ Age (SD)** | **HC Age (SD)** | **Education SCZ nr. (SD)** | **Education HC nr. (SD)** | **CPZ eq. (SD)** | **SCZ status** | **Inpatients/**  **Outpatients** | **Illness Duration mean (SD)** | **DUP mean (SD)** | **Age at Onset mean (SD)** | **PANSS Total mean (SD)** | **PANSS Positive mean (SD)** | **PANSS negative mean (SD)** | **GAF** | **Diagnostic Criteria** |
| --- | --- | --- | --- | --- | --- | --- | --- | --- | --- | --- | --- | --- | --- | --- | --- | --- | --- | --- | --- |
| Yoon 2012 | 51 | 51 | 12 (23.53) | 25 (49.02) | 19.92 (3.59) | 20.22 (3.90) | 12.32  (2.34) | 14.00  (2.94) | NA | FE | NA | NA | NA | NA | NA | NA | NA | 47.75  (9.97) | DSM-IV-TR |
| Pettersson-Yeo 2013(b) | 19 | 19 | 7 (36.84) | 7 (36.84) | 24.37 (4.71) | 24.89 (4.41) | NA | NA | 204.08 (116.35) | FE | NA | < 2 | NA | NA | 54.37  (15.13) | 12.58  (3.96) | 13.79  (5.26) | NA | DSM-IV |
| Rish 2013 | 11 | 11 | NA | NA | NA | NA | NA | NA | all SSD taking AP | Chronic, pharmaco-resistant | NA | NA | NA | NA | NA | NA | NA | NA | DSM-IV-TR |
| Castro 2014 | 31 | 21 | 10 (32.26) | 13 (61.90) | 27.70 (8.20) | 26.60 (7.40) | NA | NA | NA | NA | NA | NA | NA | NA | NA | NA | NA | NA | DSM-IV-TR |
| Bendfeldt 2015 | 19 | 19 | NA | NA | NA | NA | NA | NA | NA | FE | NA | NA | NA | NA | NA | NA | NA | 54.95 (17.00) | PACE, ICD-10 |
| Gaebler 2015 | 24 | 24 | 10 (41.67) | 10 (41.67) | 36.10 (9.20) | 36.40 (9.30) | 16.30  (3.00) | 15.00  (2.50) | 156.90 (77.30) | Chronic | NA | 8.30  (8.20) | NA | NA | 43.30  (9.40) | 10.40  (3.00) | 11.50  (4.10) | NA | DSM-IV, ICD-10 |
| Koch 2015 | 44 | 44 | 17 (38.64) | 9 (20.45) | 34.20 (9.80) | 37.10 (10.90) | NA | NA | NA | Chronic | NA | 4.80  (5.60) | NA | NA | 85.70  (27.20) | NA | NA | NA | DSM-IV, ICD-10 |
| Bae 2018 | 21 | 54 | 6 (28.57) | 21 (38.89) | 24.26 (3.74) | 22.51 (4.49) | 12.13  (1.87) | 12.98  (3.05) | NA | NA | Outpatient | NA | NA | NA | NA | NA | NA | NA | DSM-IV |
| Juneja 2018 | 34 | 34 | NA | NA | 39.76 (10.81) | 37.88 (12.29) | NA | NA | NA | NA | NA | NA | NA | NA | NA | NA | NA | NA | DSM-IV |
| Juneja 2018(a) | 25 | 25 | NA | NA | 44.48 (10.77) | 40.08 (11.35) | NA | NA | NA | NA | NA | NA | NA | NA | NA | NA | NA | NA | DSM-IV |
| Orban 2018 | 191 | 191 | 52 (27.00) | 48 (25.00) | NA | NA | NA | NA | NA | NA | NA | NA | NA | NA | NA | NA | NA | NA | NA |
| Viviano 2018 | 113 | 75 | 37 (32.74) | 33 (44.00) | 34.1 (10.10) | 31.3 (10.10) | 13.21 (NA) | 15.60 (NA) | NA | NA | NA | NA | NA | NA | NA | NA | NA | NA | DSM IV / DSM-IV-TR |
| Oh 2019 | 103 | 41 | 46 (44.66) | 17 (41.46) | 32.46 (9.21) | 33.98 (8.53) | 13.59  (2.76) | 16.22  (2.57) | NA | NA | NA | 4.69  (4.25) | NA | 27.20  (9.00) | 49.93  (18.98) | 13.09  (6.70) | 12.09  (5.48) | NA | DSM-IV-TR |
| Antonucci 2020 | 65 | 65 | 15 (23.08) | 12 (18.46) | 29.00 (6.00) | 29.00 (5.00) | NA | NA | 678.50 (351.31) | NA | NA | NA | NA | NA | 98.44  (29.3) | NA | NA | NA | DSM-IV |
| Yang 2020 | 236 | 150 | NA | NA | 22.01 (6.50) | 23.66 (5.60) | 11.50  (3.68) | 14.05  (4.34) | 203.52 (184.56) | NA | NA | 1.96  (3.24) | 1.38  (NA) | NA | NA | NA | NA | NA | DSM-IV |
| Ghosal 2021 | 53 | 53 | NA | NA | NA | NA | NA | NA | NA | NA | NA | NA | NA | NA | NA | NA | NA | NA | NA |
| Potvin 2021 | 39 | 42 | 19 (48.72) | 22 (52.38) | 32.50 (6.90) | 30.50 (8.50) | 11.60  (3.10) | 17.90  (3.70) | 378.00 (208.00) | Chronic | NA | 9.80  (7.10) | NA | NA | 80.90  (13.80) | 18.90  (6.70) | 20.80  (6.90) | NA | DSM-IV |
| Sheffield 2021 | 29 | 31 | 7 (24.14) | 8 (25.81) | 37.79 (12.47) | 34.69 (10.47) | 12.69  (3.65) | 16.55 (3.05) | NA | NA | NA | NA | NA | NA | NA | NA | NA | NA | DSM-5 |

FE: first-episode.

**Tab. S5 – Multimodal MRI studies – Samples Characteristics.**

| **Author, Year** | **SCZ nr.** | **HC nr.** | **SCZ F nr. (%)** | **HC F nr. (%)** | **SCZ Age (SD)** | **HC Age (SD)** | **Education SCZ nr.**  **(SD)** | **Education HC nr.**  **(SD)** | **CPZ eq.**  **(SD)** | **SCZ status** | **Inpatients/**  **Outpatients** | **Illness Duration mean (SD)** | **DUP mean (SD)** | **Age at Onset mean**  **(SD)** | **PANSS Total mean (SD)** | **PANSS Positive mean (SD)** | **PANSS negative mean (SD)** | **GAF** | **Diagnostic Criteria** |
| --- | --- | --- | --- | --- | --- | --- | --- | --- | --- | --- | --- | --- | --- | --- | --- | --- | --- | --- | --- |
| Venkataraman 2010 | 18 | 18 | 0 (0) | 0 (0) | NA | NA | NA | NA | NA | Chronic | NA | NA | NA | NA | NA | NA | NA | NA | NA |
| Du 2012 | 28 | 28 | 5 (17.86) | 9 (32.14) | 39.40 (12.70) | 31.50 (11.10) | NA | NA | NA | Chronic | NA | NA | NA | NA | NA | 15.80  (5.50) | 15.40  (5.60) | NA | NA |
| Lee 2018 | 47 | 23 | 29 (61.70) | 15 (65.22) | 28.68 (6.23) | 29.70 (5.15) | NA | NA | 296.59 (2657.34) | NA | NA | 1.02  (1.58) | NA | NA | 61.11  (14.92) | 15.91  (6.51) | 16.77  (7.08) | NA | DSM-IV-TR |
| Liu 2018(a) | 62 | 33 | 0 (0) | 0 (0) | 47.03 (6.22) | 45.12 (10.01) | 8.94  (1.97) | 10.67  (2.79) | NA | NA | Inpatients | NA | NA | NA | NA | NA | NA | NA | NA |
| Liang 2019 | 98 | 106 | 41 (41.84) | 54 (50.94) | 24.75 (7.74) | 25.97 (8.47) | NA | NA | 27/98 taking AP for <3days | FE | NA | 1.49  (2.09) | 1.49  (2.09) | NA | 93.49  (20.16) | NA | NA | NA | DSM-IV |
| Liang 2019(a) | 54 | 48 | 36 (66.67) | 32 (66.67) | 28.40 (7.06) | 25.25 (7.22) | NA | NA | 10/54 taking AP for <3days | FE | NA | 0.88  (1.45) | 0.88  (1.45) | NA | 92.53  (18.55) | NA | NA | NA | DSM-IV |
| Zhuang 2019 | 40 | 29 | 18 (45.00) | 15 (51.72) | 27.13 (5.90) | 27.03 (4.32) | 12.91  (3.14) | 14.21  (2.37) | 0 (0) | FE | NA | 0.54  (0.97) | 0.54  (0.97) | NA | 73.54  (17.47) | NA | NA | NA | DSM-IV |
| Bang 2021 | 86 | 79 | 55 (63.95) | 47 (59.49) | 35.12 (14.69) | 38.16 (14.92) | NA | NA | 481.69 (373.89) | NA | NA | 1.54 (3.43) | NA | NA | 109.57  (23.73) | 29.77  (9.52) | 20.74  (9.42) | NA | DSM-IV-TR / DSM V |
| Song 2022 | 191 | 191 | 100 (52.36) | 102 (53.40) | 23.16 (8.45) | 23.28 (4.69) | NA | NA | NA | NA | NA | NA | NA | NA | NA | NA | NA | NA | DSM-IV |
| Chen 2023 | 140 | 205 | 95 (67.86) | 110 (53.66) | 34.22 (8.23) | 32.51 (8.39) | 10.69  (3.32) | 12.84  (2.83) | NA | FE 61, Chronic 79 | NA | >2 | NA | NA | 85.55  (18.55) | 23.14  (5.25) | 22.53  (7.48) | NA | DSM-IV-TR |
| Gao 2023 | 89 | 118 | 0 (0) | 0 (0) | 49.18 (7.96) | 50.76 (7.60) | 8.91  (2.47) | 9.76  (2.82) | 570.17  (208.48) | NA | NA | 26.10  (8.01) | NA | 23.08  (4.48) | NA | NA | NA | NA | DSM-IV-TR |

AP: antipsychotic, FE: first-episode.

**Tab. S6 – sMRI studies – Model Setting.**

| **Author, Year** | **Scan** | **Algorithm** | **Brain Features** | **Features Reduction** | **Features Selection** | **Validation** | **BAC** | **Sensitivity** | **Specificity** | **AUC** |
| --- | --- | --- | --- | --- | --- | --- | --- | --- | --- | --- |
| Davatzikos 2005 | 1.5 T | SVM | RAVENS density maps | / | / | LOOCV | 0.81 | 0.74 | 0.87 | NA |
| Yushkevich 2005 | 1.5 T | SVM | VOIs | ROIs (various methods of parcellations) | Energy-based feature selection L2-regularization | LOOCV | 0.71 | 0.72 | 0.7 | NA |
| Kawasaki 2007 | 1.5 T | LDA | GMV | / | ANCOVA | LOOCV | 0.9 | 0.9 | 0.9 | NA |
| Kawasaki(a) 2007 | 1.5 T | LDA | GMV | / | Not applicable / Consistency with discovery set | External validation | 0.84 | 0.88 | 0.81 | NA |
| Santos 2010 | 1.5 T | Maximum uncertainty LDA | Structural images intensity | PCA | / | LOOCV | 0.65 | 0.7 | 0.6 | NA |
| Takayanagi 2011 | 1.5 T | LDA | CT | ROIs (DD atlas) | Stepwise feature selection | Held out (70:30) | 0.86 | 0.89 | 0.83 | NA |
| Takayanagi 2011(a) | 1.5 T | LDA | CT | ROIs (DD atlas) | Stepwise feature selection | Held out (70:30) | 0.83 | 0.67 | 1 | NA |
| Wang 2011 | 1.5 T | SVM | ICs of GM densities | PCA | A priori (mask derived from literature) Thresholding | 10-fold CV | 0.83 | 0.91 | 0.75 | NA |
| Castellani 2012 | 1.5 T | SVM | Feature vocabulary | ROIs | A priori (DLPFC) | LOOCV | 0.77 | 0.82 | 0.72 | 0.79 |
| Ota 2012 | 1.5 T | LDA | GMV and CSFV | / | Not applicable / Consistency with discovery set | External validation | 0.72 | 0.74 | 0.7 | NA |
| Pettersson-Yeo 2013 | 3T | SVM | GM images | / | / | LOOCV | 0.63 | 0.58 | 0.68 | NA |
| Gould 2014 | 1.5 T | SVM | GMV + WMV | ROIs (Harvard–Oxford cortical and subcortical, Probabilistic cerebellar, Talairach, JHU WM tractography atlas) | A priori (thalamus, putamen, pallidum left and right cerebellar) | Leave-two-out CV | 0.69 | 0.67 | 0.7 | NA |
| Radulescu 2014 | 1.5T | LDA | GM heterogeneity and GM volume | PCA | ANCOVA T-test | LOOCV Held out (70:30) | 0.73 | 0.71 | 0.75 | NA |
| Guo 2016 | 3T | SVM | CT | ROIs (Destrieux sulcogyral atlas) | / | LOOCV | 0.82 | 0.78 | 0.87 | NA |
| Ou 2016 | 3T | SVM | WMV | / | / | LOOCV | 0.83 | 0.82 | 0.84 | NA |
| Yun 2016 | 3T | SVM | CSA-ISC | ROIs (Destrieux atlas) | T-test | LOOCV | 0.92 | 0.91 | 0.93 | NA |
| Dluhos 2017 | 1.5T/3T | SVM | GM density (meta-model) | / | / | LOOCV Leave-one-site-out CV | 0.66 | 0.66 | 0.67 | NA |
| Liu 2017 | 3T | SVM | Structural brain hierarchical network | ROis (cortical AAL atlas) | mRMR SVM-RFE | Nested 6x6-fold CV | 0.91 | 0.87 | 0.94 | 0.95 |
| Salvador 2017 | 1.5T | LR | GMV, WMV, CT, Regional volumes and pairwise interactions | ROIs (Desikan atlas) PCA | T-test L0-regularization | Nested 10x10-fold CV | 0.79 | 0.84 | 0.75 | NA |
| Rozycki 2018 | 1.5T/3T | SVM | RAVENS maps, regional volumes, region-based descriptors | ROIs | / | 10-fold CV | 0.77 | 0.84 | 0.7 | 0.84 |
| Schwarz 2019 | 1.5T/3T | SVM | GM density | ROIs (AAL atlas) | / | 10-fold CV Leave-one-site-out CV | 0.76 | 0.75 | 0.76 | 0.81 |
| Winterburn 2019 | 1.5T | LDA | CT | PCA | / | 10-fold CV Held out (2:1) | 0.68 | 0.66 | 0.71 | NA |
| Winterburn 2019(a) | 3T | SVM | CT | PCA | / | 10-fold CV Held out (2:1) | 0.74 | 0.59 | 0.88 | NA |
| Chang 2020 | 3T | EDNN | WMV | / | Kullback-Leibler-L1 regularization | Held out (87.5:12.5) | 0.9 | 0.89 | 0.91 | 0.96 |
| Chang 2020(a) | 3T | EDNN | WMV | / | Kullback-Leibler-L1 regularization | Held out (70:30) | 0.84 | 0.97 | 0.72 | 0.97 |
| Lei 2020 | 3T | SVM | Structural covariance matrix, GMV, WMV | ROIs (AAL90 atlas) | / | Nested 10-fold CV | 0.87 | 0.75 | 0.99 | NA |
| Oh 2020 | 1.5T | CNN | Converted video of a subject’s structural MR images | / | Not applicable / Consistency with discovery set | External validation | 0.71 | 0.88 | 0.54 | 0.72 |
| Vieira 2020 | 3T | DNN | Surfaced-based regional volumes and CT | ROIs (DK atlas) PCA | / | Nested 10-fold CV | 0.7 | 0.7 | 0.7 | NA |
| Vieira 2020(a) | 1.5T | LR | Voxel-based CT | PCA | / | Nested 10-fold CV | 0.6 | 0.57 | 0.62 | NA |
| Yamamoto 2020 | 3T | SVM | GMV | Mask for densitiy threshold | / | 10-fold CV Leave-one-site-out CV | 0.72 | 0.62 | 0.83 | NA |
| Li 2021 | NA | CNN | GMV | / | / | 10-fold CV | 1 | 1 | 1 | 1 |
| Lieslehto 2021 | 1.5T | SVM | GMV | PCA | Not applicable / Consistency with discovery set | External validation | 0.8 | 0.76 | 0.84 | 0.87 |
| Lieslehto 2021(a) | 3T | SVM | GMV | PCA | Not applicable / Consistency with discovery set | External validation | 0.69 | 0.55 | 0.84 | 0.82 |
| Morgan 2021 | 3T | GPC | CT | ROIs (Desikan-Killiany atlas) | / | 10-fold CV | 0.63 | 0.72 | 0.52 | 0.67 |
| Zhou 2021 | 3T | SVM | CT | ROI (DK atlas) | / | LOOCV | 0.69 | 0.73 | 0.65 | NA |
| Chilla 2022 | 3T | Ensamble (SVM-RBF+Nu-SVC+LR) | Cortical and subcortical volume, CT, mean curvautre and SA | ROIs (Gaussian Classifier atlas, DK atlas) | SVC | 3-fold CV Held out (70:30) | 0.82 | 0.98 | 0.65 | 0.82 |
| Cui 2022 | 3T | DNN | GMV, WMV and CSFV | / | Ranking by Fisher scores, L1-regularization | 10-fold CV | 0.83 | 0.8 | 0.85 | 0.87 |
| Korda 2022 | 1.5T | Neural network-based classifier | Texture features (entropy, sum of entropy, difference of entropy, energy, contrast and homogeneity) | / | T-test L2-regularization | Nested 10×10-fold CV | 0.8 | 0.76 | 0.84 | NA |
| Xie 2022 | 1.5T/3T | XGBoost classifier with SWEM | MICI | ROIs (Destrieux atlas, Gaussian Classifier atlas) | / | 5-fold CV Held out (80:20) Leave-one-site-out CV (ensambled with SWEM) | 0.82 | 0.79 | 0.85 | 0.82 |
| Xie 2022(a) | 3T | XGBoost classifier with SWEM | MICI | ROIs (Destrieux atlas, Gaussian Classifier atlas) | Not applicable / Consistency with discovery set | External validation | 0.83 | 0.68 | 0.97 | 0.83 |
| Knolee 2023 | 3T | LR | Networks of GM covariance | / | ANCOVA | Held out (60:40) | 0.75 | 0.74 | 0.76 | 0.82 |
| Sun 2023 | 3T | Extra Trees | GMV | ROIs (DKT atlas) 3D-CAE | / | Nested 10-fold CV Held out CV (80:20) | 0.74 | 0.84 | 0.64 | 0.8 |
| Sun 2023(a) | 3T | Extra Trees | GMV | ROIs (DKT atlas) 3D-CAE | Not applicable / Consistency with discovery set | External validation | 0.72 | 0.8 | 0.63 | 0.77 |

AAL: automated anatomical labeling, CAE: convolutional autoencoder, CNN: convolutional neural network, CSA: cortical surface area, CSFV: cerebrospinal fluid volume, CT: cortical thickness, CV: cross-validation, DD: Desikan and Destieux, DK: Desikan-Killiany, DKT: Desikan-Killiany-Tourville, DLPFC: dorsolateral prefrontal cortex, DNN: deep neural network, EDNN: explainable deep neural network, GM: gray matter, GMV: gray matter volume, GPC: gaussian process classifier, ICs: independent components, ISC: individualized structural covariance, JHU: John Hopkins University, LDA: linear discriminant analysis, LOOCV: leave one out cross-validation, LR: logistic regression, mRMR: minimum redundancy and maximum relevance, PCA: principal component analysis, RBF: radial basis function, RFE: recursive feature elimination, ROI: region of interest, SA: surface area, SVC: support vector classifier, SVM: support vector machine, SWEM: sample-weighted ensembling model, WM: white matter, WMV: white matter volume, XGBoost: eXtreme Gradient Boosting.

**Tab. S7 – rs-fMRI studies – Model Setting.**

| **Author, Year** | **Scan** | **Algorithm** | **Brain Features** | **Features Reduction** | **Features Selection** | **Validation** | **BAC** | **Sensitivity** | **Specificity** | **AUC** |
| --- | --- | --- | --- | --- | --- | --- | --- | --- | --- | --- |
| Shi 2007 | 1.5T | Pseudo Fisher  LDA | ReHo | ROIs (AAL atlas) PCA | / | LOOCV | 0.79 | 0.83 | 0.74 | NA |
| Su 2013 | 1.5T | SVM | Functional network | ROIs (AAL atlas) | Correlation coefficient | LOOCV | 0.83 | 0.84 | 0.81 | NA |
| Anticevic 2014 | 3T | SVM | Bilateral thalamus seed-based connectivity | ROIs (Seeds) | A priori (Bilateral thalamus) | LOOCV | 0.74 | 0.76 | 0.72 | NA |
| Anticevic  2014(a) | 3T | SVM | Bilateral thalamus seed-based connectivity | / | Mask identified with the discovery sample | LOOCV | 0.73 | 0.68 | 0.78 | NA |
| Zhu 2014 | 3T | Adaptive informative vectors algorithm | Graph of functional language network | ROIs | A priori, Forward and backward feature selection | LOOCV | 0.84 | 0.82 | 0.86 | NA |
| Cheng 2015 | 1.5T/3T | SVM | BWAS | ROI (AAL atlas) | T-test | LOOCV | 0.76 | 0.77 | 0.75 | NA |
| Cheng 2015(a) | 3T | SVM | Graph networks betweenness centrality | ROIs (Shen et al. 2013) | Thresholding | LOOCV | 0.79 | 0.74 | 0.83 | NA |
| Chyzhyk 2015 | NA | SVM | fALFF | Lattice Auto-Associative Memories (LAAMs) | Correlation coefficient  Thresholding | 10-fold CV | 1 | 1 | 1 | 1 |
| Mueller 2015 | 3T | SVM | FC autonomy index | ROIs (seed) | A priori (Caudate Nucleus) | Leave-two-pair out CV | 0.73 | 0.68 | 0.78 | NA |
| Mikolas 2016 | 3T | SVM | Seed-based functional connectivity | ROIs (seed) | A priori (Anterior Insula) | leave-one-subject-per-group-out CV | 0.73 | 0.75 | 0.71 | 0.8 |
| Peters 2016 | 3T | SVM | Cortical intrinsic brain activity (Subocortical-cerebellar system and cortical networks) | ROIs (Allen et al. 2011), PCA | A priori (Subocortical-cerebellar system and cortical networks) | LOOCV | 0.91 | 0.82 | 1 | NA |
| Guo 2017 | 3T | SVM | Functional connectivity | / | ANCOVA | LOOCV | 0.94 | 0.96 | 0.93 | NA |
| Skåtun 2017 | 3T | LDA | Graph networks | / | ANOVA | LOSO-CV | 0.73 | 0.72 | 0.74 | NA |
| Kottaram 2018 | 3T | SVM | FC dynamics (space and time) | ROIs | A priori T-test Ranking (gradient descent) | 10-fold CV  Held out (6:4) | 0.91 | 0.9 | 0.92 | 0.87 |
| Liu 2018 | 3T | SVM | VMHC | / | T-test | leave-pair-out CV | 0.94 | 1 | 0.87 | NA |
| Moghimi 2018 | NA | SVM | Brain graph network | PICA ROIs (functional atlas) | Sequential Forward Selection | Nested 4x5-fold CV | 0.73 | 0.77 | 0.68 | NA |
| Ji 2019 | 3T | Correlation-based classification method | Group-level FC | ROI (Finn et al. 2015) | / | LOOCV | 0.78 | 0.78 | 0.77 | 0.84 |
| Ji 2019(a) | 3T | Correlation-based classification method | Group-level FC | ROI (Finn et al. 2015) | / | LOOCV | 0.74 | 0.8 | 0.68 | 0.86 |
| Kalmady 2019 | 3T | LR | Stacked model with ALFF, fALFF, ReHo, FC (FC-Correlation, FC-partial correlation, FC-precision) | ROIs (14 different atlas) PCA | L2-regularization | 10-fold CV | 0.86 | 0.8 | 0.93 | NA |
| Qiu 2019 | 3T | CNN | Magnitude of complex-valued ICs | / | T-test L2-regularization | 5-fold CV Held out  (60:20:20) | 0.99 | 1 | 0.98 | NA |
| Shaoqiang 2019 | 3T | SVM | Network-based FC | ROI (AAL atlas) | A priori (12 brain networks) | 10-fold CV | 0.83 | 0.92 | 0.75 | 0.9 |
| Zhu 2019 | 3T | SVM | PAS | / | ANCOVA | LOOCV | 0.75 | 0.68 | 0.82 | NA |
| Cai 2020 | 3T | LDA | Spatial ICs | Kernel PCA | T-test Fisher LDA | LOOCV | 0.71 | 0.63 | 0.78 | NA |
| Cai 2020(a) | 3T | LDA | Spatial ICs | / | Not applicable / Consistency with discovery set | External validation | 0.71 | 0.64 | 0.78 | NA |
| Cao 2020 | 3T | SVM | Mutual information FC | ROI (DK atlas) | / | LOOCV | 0.79 | 0.74 | 0.83 | NA |
| Ji 2020 | 3T | SVM | ReHo | ROIs (Stanford University functional brain atlas) | / | 20-fold CV  Held out (80:20) | 0.77 | 0.7 | 0.85 | 0.69 |
| Liang 2020 | 3T | GBDT | Seed-based (voxel-wise FC) | ROIs (Seed) | A priori (PCC) | 5-fold CV Held out (80:20) | 0.73 | 0.74 | 0.73 | 0.73 |
| Liang 2020(a) | 3T | GBDT | Seed-based (region-wise FC) | / | Not applicable / Consistency with discovery set | External validation | 0.73 | 0.61 | 0.85 | 0.73 |
| Sartipi 2020 | 3T | SVM | ICs amplitude | PCA Orthogonal Ripplet-II Transform  OLPP | T-test | 5-fold CV | 1 | 1 | 1 | 1 |
| Wang 2020 | 3T | SVM | fALFF at different frequency bands | / | H-TS learning  L1,1-regularization | LOOCV | 0.93 | 0.96 | 0.91 | 0.97 |
| Wang 2020(a) | 3T | SVM | FC strength | / | Correlation coefficient | 10-fold CV | 0.8 | 0.79 | 0.81 | NA |
| Yang 2020 | 3T | SVM | Brain activity synchronization | / | Correlation coefficient | 5-fold CV Held out (84:16) | 0.95 | 1 | 0.9 | NA |
| Yoshihara 2020 | 3T | Sparse LR | FC | ROIs (Brainvisa Sulci atlas, AAL atlas) | L1-regularized SCCA | LOOCV | 0.76 | 0.72 | 0.79 | 0.83 |
| Yoshihara 2020(a) | 3T | Sparse LR | FC | / | Not applicable / Consistency with discovery set | External validation | 0.61 | 0.64 | 0.58 | 0.66 |
| Yoshihara 2020(b) | 3T | Sparse LR | FC | / | Not applicable / Consistency with discovery set | External validation | 0.44 | 0.4 | 0.47 | 0.42 |
| Lyu 2021 | 3T | PNN | Causal EC - GC | ROIs | A priori (Middle occipital gyrus and Insula) | LOOCV | 0.95 | 0.93 | 0.97 | 0.98 |
| Serin 2021 | 1.5T | DT | Brain graph network | ROIs (AAL atlas) | Suprathreshold edge selection | 10-fold CV | 0.89 | 0.92 | 0.87 | NA |
| Wang 2021 | 3T | CNN | Brain network | / | / | 5-fold CV | 0.84 | 0.79 | 0.89 | 0.84 |
| Wiem 2021 | 3T | Deep CNN | Bold signal time series | ROIs (AAL atlas) | Deep Convolutional CTN, GLP | Held out (85:15) | 0.99 | 0.99 | 1 | NA |
| Feng 2022 | 3T | SVM | Cerebellar network FC | ROIs (Buckner et al. 2011) | Correlation coefficient, Thresholding | 10-fold-CV | 0.74 | 0.68 | 0.8 | 0.79 |
| Gao 2022 | 3T | SVM | ALFF | / | T-test | LOOCV | 0.73 | 0.92 | 0.55 | 0.72 |
| Gao 2022(a) | 3T | SVM | fALFF | / | T-test | LOOCV | 0.98 | 0.98 | 0.98 | NA |
| Lee 2022 | 3T | SVM | FC | ROIs (AAL3 atlas) | / | Nested 10x10-fold CV | 0.85 | 0.87 | 0.83 | 0.92 |
| Lei 2022 | 3T | GCN | Graph networks | ROIs (AAL atlas) | L2-regularization | 10-fold CV | 0.86 | 0.74 | 0.98 | 0.79 |
| Oh 2022 | 3T | CNN | Brain graph network | ROIs (AAL atlas) | L1-regularization | Nested 10-fold CV | 0.83 | 0.86 | 0.8 | 0.89 |
| Yang 2022 | 3T | SVM | CAP in four frequency sub-bands | ROIs (Schaefer et al., 2018; Yang et al., 2021) | / | Leave-one-pair-out (LOPO) CV | 0.9 | 0.92 | 0.89 | 0.95 |
| Zhao 2022 | 3T | HDLFCA | ICs time courses + FC | PCA | / | Nested 10x3 fold CV | 0.85 | 0.87 | 0.83 | 0.92 |
| Ellis 2023 | 3T | CNN | Dynamic FNC | / | / | 10-fold stratified shuffle split CV (80-10-10) | 0.75 | 0.74 | 0.76 | NA |
| Hancock 2023 | 3T | Naïve Bayes classifier | Mode-metastability of FC | ROIs (AAL atlas) | / | 10-fold CV | 0.63 | 0.66 | 0.6 | 0.68 |
| Huang 2023 | 3T | CNN | Static and a dynamic pathway of functional neural network | ROIs (AAL atlas) | / | 5-fold-CV | 0.82 | 0.81 | 0.82 | NA |
| Kang 2023 | 3T | SVM | RQA and cross-RQA | PCA, ROIs (DMN network templates) | RFE | Nested 10-fold CV | 0.77 | 0.75 | 0.78 | 0.82 |
| Li 2023 | 3T | SVM | Functional networks | / | / | 2-fold CV | 0.77 | 0.76 | 0.79 | 0.82 |
| Ma 2023 | 3T | SVM | fALFF, ReHo, FC | / | ANCOVA | LOOCV | 0.88 | 0.9 | 0.86 | NA |
| Ma 2023(a) | 3T | SVM | fALFF, ReHo, FC | / | Not applicable / Consistency with discovery set | External validation | 0.88 | 0.88 | 0.89 | NA |
| Xing 2023 | NA | SVM | FC | / | WNRE | 10-fold CV | 0.84 | 0.85 | 0.83 | NA |

AAL: automated anatomical labeling , ALFF: amplitude of low-frequency fluctuations, BWAS: brain-wide association, CAP: co-activation pattern, CNN: convolutional neural network, CTN: curvelet transform network, CV: cross-validation, DK: Desikan-Killiany, DMN: default mode network, DT: decision tree, EC: effective connectivity, fALFF: fractional amplitude of low-frequency fluctuations, FC: functional connectivity, FNC: functional network connectivity, GBDT: gradient boosting decision tree, GC: granger causality, GCN: graph convolutional network, GLP: geometric Lp-norm pooling, H-TC: Hierarchical structured sparse , HDLFCA: hybrid learning framework integrating brain connectivity and activity, ICs: independent components, LDA: linear discriminant analysis, LOOCV: leave one out cross-validation, OLPP: orthogonal locality preserving projection, PAS: parameter of asymmetry, PCA: principal component analysis, PCC: posterior cingulate cortex, PICA: Probabilistic independent component analysis, PNN: probabilistic neural network, ReHo: regional homogeneity, RFE: recursive feature elimination, RQA: recurrence quantification analysis, ROI: region of interest, SCCA: sparse canonical correlation analysis, SVC: support vector classifier, SVM: support vector machine, VMHC: voxel-mirrored homotopic connectivity, WNRE: weighted neighborhood rough set combined with entropy.

**Tab. S8 – DTI studies – Model Setting.**

| **Author, Year** | **Scan** | **Algorithm** | **Brain Features** | **Features Reduction** | **Features Selection** | **Validation** | **BAC** | **Sensitivity** | **Specificity** | **AUC** |
| --- | --- | --- | --- | --- | --- | --- | --- | --- | --- | --- |
| Rathi 2010 | NA | k-NN | PDF | / | / | Leave many-out CV | 0.86 | 0.86 | 0.85 | NA |
| Ardekani 2011 | 1.5T | LDA | MD | PCA | T-test | Held out (50:50) | 0.98 | 0.96 | 1 | NA |
| Petterson-Yeo 2012(a) | 3T | SVM | FA skeletons | / | / | LOOCV | 0.66 | 0.68 | 0.63 | NA |
| Zheng 2013 | 3T | SVM | Structural connectivity network | ROIs (AAL) | L1-regularization | Held out (50:50) | 0.97 | 0.96 | 0.98 | NA |
| Chen 2018 | 3T | PLS-based classification | Generalized FA | ROIs (TBAA) | A priori (76 fiber tract bundles) | Held out (70:30) | 0.76 | 0.75 | 0.76 | 0.79 |
| Mikolas 2018 | 3T | SVM | FA | / | / | leave-two-out CV | 0.62 | 0.60 | 0.65 | NA |
| Deng 2019 | 3T | RF | FA, RD, AD and MD | ROIs (TBAA) | RFE | LOOCV  Held out (80:20) | 0.76 | 0.77 | 0.75 | 0.74 |
| Huang 2020 | 3T | k-NN | Node distance matrix | ROIs-based Fiber tracking (AAL atlas), t-SNE | / | LOOCV | 0.78 | 0.83 | 0.72 | NA |
| Lin 2021 | 3T | SVM | Anatomical networks | ROI (Human Brainnetome Atlas) | T-test | Nested CV (LOOCV + 10-fold CV) | 0.89 | 0.86 | 0.92 | NA |
| Masouidi 2021 | 3T | DBN | FA | ROIs (AAL atlas) | / | 10-fold CV | 0.66 | 0.68 | 0.63 | NA |
| Morgan 2021(a) | 3T | GPC | MD | ROIs (DK atlas) | / | 10-fold CV | 0.77 | 0.65 | 0.86 | 0.86 |
| Morgan 2021(b) | 3T | GPC | Structural connectivity | ROIs (DK atlas) | / | 10-fold CV | 0.56 | 0.62 | 0.47 | 0.59 |
| Wang 2022 | 3T | LR | GM, WM, and functional brain networks | ROIs (268-node functional atlas) | Sparsity threshold, RFE | 10-fold CV Held out (4:1) | 0.66 | 0.57 | 0.76 | 0.69 |

AAL: automated anatomical labeling, AD: axial diffusivity, CSFV: cerebrospinal fluid volume, CT: cortical thickness, CV: cross-validation, DBN: deep belief network, DK: Desikan-Killiany, GM: gray matter, GMV: gray matter volume, GPC: gaussian process classifier, FA: fractional anisotropy, k-NN: k-nearest neighbors, LDA: linear discriminant analysis, LR: logistic regression, LOOCV: leave one out cross-validation, MD: mean diffusivity, MICI: Morphometric integrated classification index, mRMR: minimum redundancy and maximum relevance, PCA: principal component analysis, PDF: probability density function, PLS: patient-like-score, RD: radial diffusivity, RF: random forest, RFE: recursive feature elimination, ROI: region of interest, SVC: support vector classifier, SVM: support vector machine, t-SNE: t-stochastic neighbor embedding, TBAA: tract-based automatic analysis, VOI: volume of interest, WM: white matter, WMV: white matter volume.

**Tab. S9 – Task-fMRI studies – Model Setting.**

| **Author, Year** | **Scan** | **Task** | **Algorithm** | **Brain Features** | **Features Reduction** | **Features Selection** | **Validation** | **BAC** | **Sensitivity** | **Specificity** | **AUC** |
| --- | --- | --- | --- | --- | --- | --- | --- | --- | --- | --- | --- |
| Yoon 2012 | 1.5 T | AX-CPT | LDA | Brain Activation | ROIs | A priori (DLPFC) | LOOCV | 0.62 | 0.59 | 0.65 | NA |
| Pettersson-Yeo 2013(b) | 3T | HSCT | SVM | Brain Activation | / | / | LOOCV | 0.68 | 0.63 | 0.74 | NA |
| Rish 2013 | 1.5T | Auditory stimuli | SVM | Functional networks properties | ROIs | A priori (10 ROIs based on previous literature)  T-test | LOOCV | 0.82 | 0.82 | 0.82 | NA |
| Castro 2014 | 1.5 T | AOD | ν-MKL | Magnitude and phase of ICs | PCA ROIs (158 cubical regions) | Thresholded active voxels RFE-SVM L2-regularization | Nested LOOCV | 0.83 | 0.91 (average) | 0.75 (average) | NA |
| Bendfeldt 2015 | 3T | N-back | SVM | Brain Activation | / | / | LOOCV | 0.66 | 0.74 | 0.58 | NA |
| Gaebler 2015 | 3T | Auditory mismatch | SVM | Task-based connectivity | ROIs (AAL atlas) | Correlation coefficient | LOOCV | 0.9 | 0.88 | 0.92 | NA |
| Koch 2015 | 1.5T | Monetary incentive delay | SVM | Brain Activation | / | Searchlight | LOOCV | 0.93 | 0.98 | 0.87 | NA |
| Bae 2018 | 3 T | N-back | SVM | Graph networks | ROIs (AAL atlas) | Tresholding Relief alghoritm | 10-fold CV | 0.92 | 0.92 | 0.92 | NA |
| Juneja 2018 | 1.5T | AOD | SVM | 3-D spatial maps (ICs score-map) | PCA Generalized discriminant analysis | Novel fuzzy rough feature selection | LOOCV | 0.97 | 0.94 | 1 | NA |
| Juneja 2018(a) | 3T | AOD | SVM | 3-D spatial maps (ICs score-map) | PCA Generalized discriminant analysis | Novel fuzzy rough feature selection | LOOCV | 0.98 | 1 | 0.96 | NA |
| Orban 2018 | 1.5T/3T | Various | SVM | Task-based connectivity | ROIs (64 nodes) | / | 10-fold CV | 0.66 | 0.62 | 0.69 | NA |
| Viviano 2018 | 3T | Imitate/Observe | SVM | Task-based connectivity, brain activation | ROIs (268-node functional atlas)  Hierarchical clustering | Correlation coefficient | 10-fold CV | 0.87 | 0.89 | 0.84 | 0.89 |
| Oh 2019 | 3T | Using negative and neutral images to IORs | CNN | Brain Activation | / | / | 10-fold CV | 0.84 | 0.88 | 0.80 | 0.90 |
| Antonucci 2020 | 3T | VAC | SVM | Task-based connectivity (Stacking-based classifiers) | ROIs (Dosenbach atlas) Pruned of zero-variance features PCA | Forward feature selection | Nested 10x10-fold CV | 0.67 | 0.62 (average) | 0.72 (average) | NA |
| Yang 2020 | 3T | 2-back | SVM | Graph network | ROIs (Power atlas) | Thresholded network densities ANOVA | 5-fold CV | 0.71 | 0.86 | 0.55 | 0.7 |
| Ghosal 2021 | 3T | N-Back | LR | Brain Activation | ROIs (Brainnetome atlas) Dictionary Learning | L2,1-regularization | 10-fold CV | 0.59 | 0.62 | 0.55 | 0.63 |
| Potvin 2021 | 3 T | Shepard and Metzler’s mental rotation | LR | Task-based connectivity | ROIs (Harvard-Oxford atlas) | A priori (14 ROIs) t-test L1-regularization | LOOCV Held out (75:25) | 0.86 | 0.83 | 0.89 | 0.86 |
| Sheffield 2021 | 3T | RITL | SVM | Task-based connectivity | ROIs (Power atlas) | A priori (10 functional networks) | Leave-two-subjects-out CV | 0.80 | 0.79 | 0.81 | NA |

AAL: automated anatomical labeling, AOD: auditory oddball, AX-CPT: AX continuous performance task, CNN: convolutional neural network, CV: cross-validation, DLPFC: dorsolateral prefrontal cortex, HSCT: hayling sentence completion task, ICs: independent components, IOR: ideas of reference, LDA: linear discriminant analysis, LOOCV: leave one out cross-validation, LR: logistic regression, MKL: multiple kernel learning, PCA: principal component analysis, RFE: recursive feature elimination, RITL: rapid instructed task learning, ROI: region of interest, SSAE: stacked sparse autoencoder SVC: support vector classifier, SVM: support vector machine, VAC: variable attentional control.

**Tab. S10 – Multimodal MRI studies – Model Setting.**

| **Author, Year** | **Scan** | **Data Modality** | **Algorithm** | **Brain Features** | **Features Reduction** | **Features Selection** | **Validation** | **BAC** | **Sensitivity** | **Specificity** | **AUC** |
| --- | --- | --- | --- | --- | --- | --- | --- | --- | --- | --- | --- |
| Venkataraman 2010 | 3T | DTI + rs-fMRI | SVM | Structural and functional connectivity network | ROIs | A priori (8 brain structures) | 10-fold CV | 0.64 | 0.6 | 0.67 | NA |
| Du 2012 | 1.5T/3T | rs-fMRI + task-fMRI  (AOD task) | Combination-based Majority Vote Classifier | Amplitude of ICs | PCA | T-test Fisher LDA | LOOCV | 0.99 | 0.98 | 1 | NA |
| Lee 2018 | 3T | DTI + sMRI | SVM | GMV, FA and Trace | ROI (Desikan-Killiany atlas) | mRMR | LOOCV | 0.92 | 0.9 | 0.95 | NA |
| Liu 2018(a) | 3T | DTI + sMRI | MFMK-SVM | Cortical GMV, CT, MD, FA | ROIs (DK, Destrieux atlas, ICBM-DTI-81 atlas,  JHU white-matter tractography atlas) | mRMR SVM-RFE | 6-fold CV | 0.92 | 0.91 | 0.92 | 0.95 |
| Liang 2019 | 3T | DTI + sMRI | GBDT | Cortical GMV, CT, local GI, FA, MD | ROIs (DK, JHU DTI-based WM atlas) | Gini importance Feature fusion | 10-fold CV | 0.75 | 0.77 | 0.73 | 0.75 |
| Liang 2019(a) | 3T | DTI + sMRI | GBDT | Cortical GMV, CT, local GI, FA, MD | Mask | Features selected in Discovery Set | 10-fold CV | 0.78 | 0.81 | 0.75 | 0.78 |
| Zhuang 2019 | 3T | DTI + rs-fMRI + sMRI | MKL-SVM | CT, GMV, SA, MC, CI, FI, FA, MD, FC, fALFF | ROIs (Harvard–Oxford cortical and subcortical, Probabilistic cerebellar, JHU white-matter tractography atlas, Harvard–Oxford subcortical, Talairach atlas) | A priori (thalamus, putamen, left and right cerebellar) L1-regularized sparse coding (SC) | 10-fold CV | 0.83 | 0.93 | 0.73 | 0.82 |
| Bang 2021 | 3T | DTI + sMRI | Extra Trees | Radiomic features | ROIs | A priori (corpus callosum) Mutual information algorithm | 10-fold CV Held out (70:30) | 0.8 | 0.83 | 0.77 | 0.89 |
| Song 2022 | 3T | rs-fMRI + sMRI | SVM | dALFF + GMV | ROIs (AAL atlas) | A priori (15 Brain Networks) | 10-fold CV | 0.71 | 0.7 | 0.71 | 0.77 |
| Chen 2023 | 3T | rs-fMRI + sMRI | GCN | Multimodal brain graphs network | ROIs (268-node functional atlas) | Sparsity threshold RFE | Held out (85:15) | 0.96 | 0.95 | 0.96 | 0.96 |
| Gao 2023 | 3T | rs-fMRI + sMRI | SVM | GMV, fALFF, ReHo | / | / | 10-fold CV | 0.89 | 0.82 | 0.96 | 0.96 |

AAL: automated anatomical labeling, CI: curvature index, CT: cortical thickness, CV: cross-validation, dALFF: dynamic fractional amplitude of low-frequency fluctuation, DK: Desikan-Killiany, GBDT: gradient boosting decision tree, GCN: graph convolutional network, GMV: gray matter volume, LOOCV: leave one out cross-validation, FA: fractional anisotropy, fALFF: fractional amplitude of low-frequency fluctuations, FC: functional connectivity, FI: folding index, GI: gyrification index, ICs: independent components, LDA: linear discriminant analysis, MC: mean curvature, MD: mean diffusivity, MFMK: multiple feature multiple kernel, MKL: multiple kernel learning, mRMR: maximum relevance minimum redundancy JHU: John Hopkins University, PCA: principal component analysis, ReHo: regional homogeneity, RFE: recursive feature elimination, ROI: region of interest, SA: surface area, SVM: support vector machine

**Fig. S1 - Forest Plot – Sensitivity Fig.
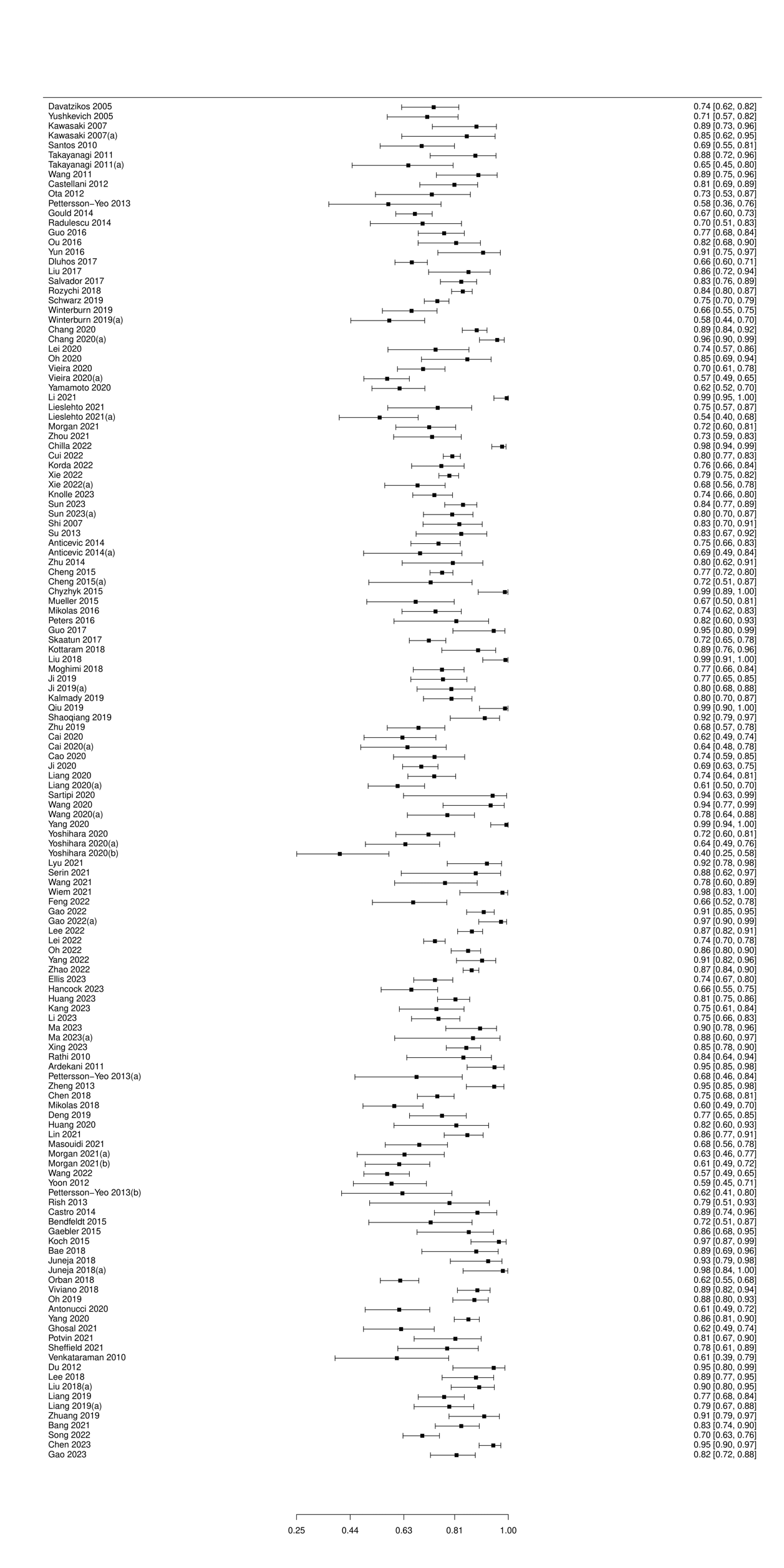
S2 - Forest Plot – Specificity
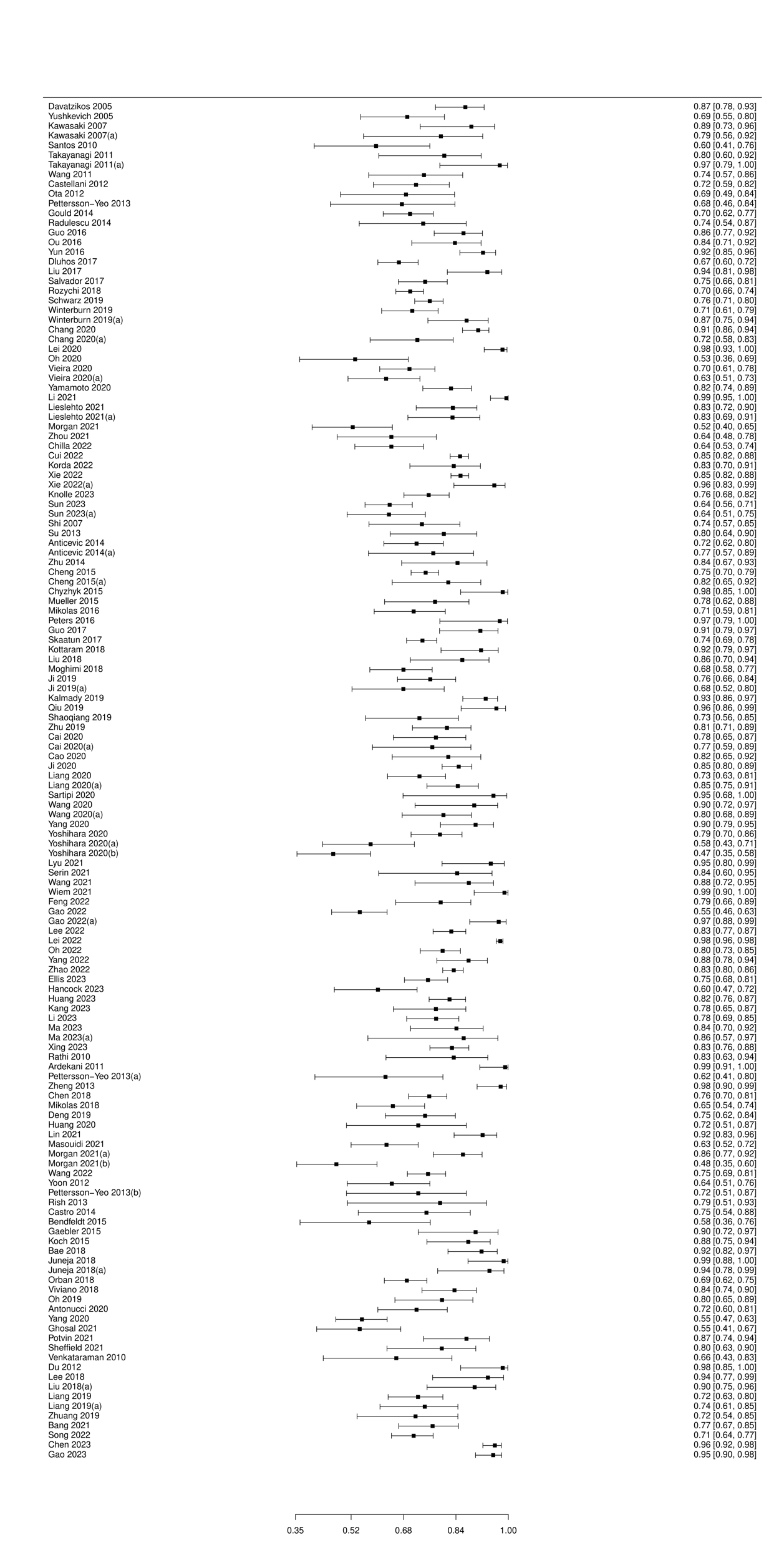
**

**
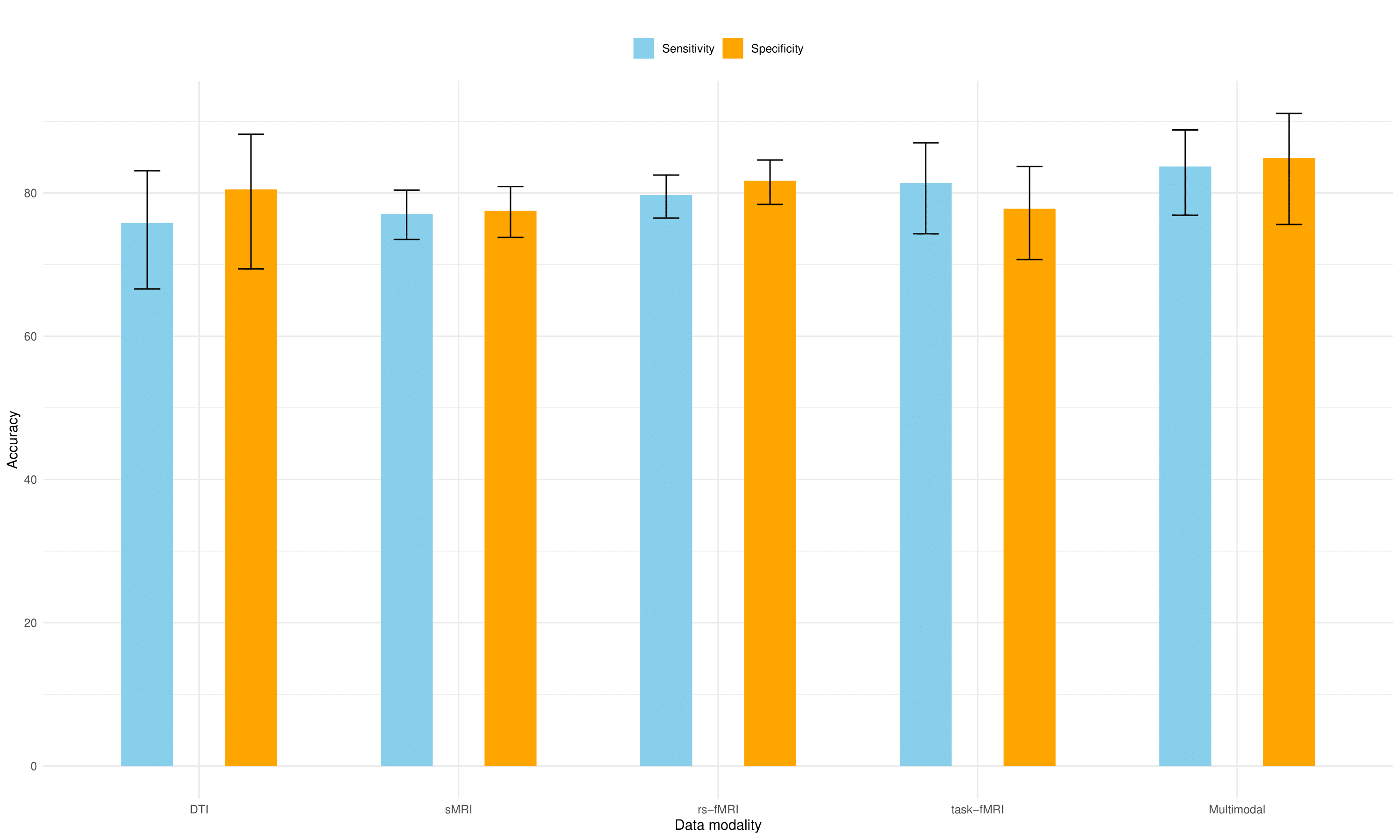
**

**Fig. S3 – Summary of classification accuracy for each data modality.**

**
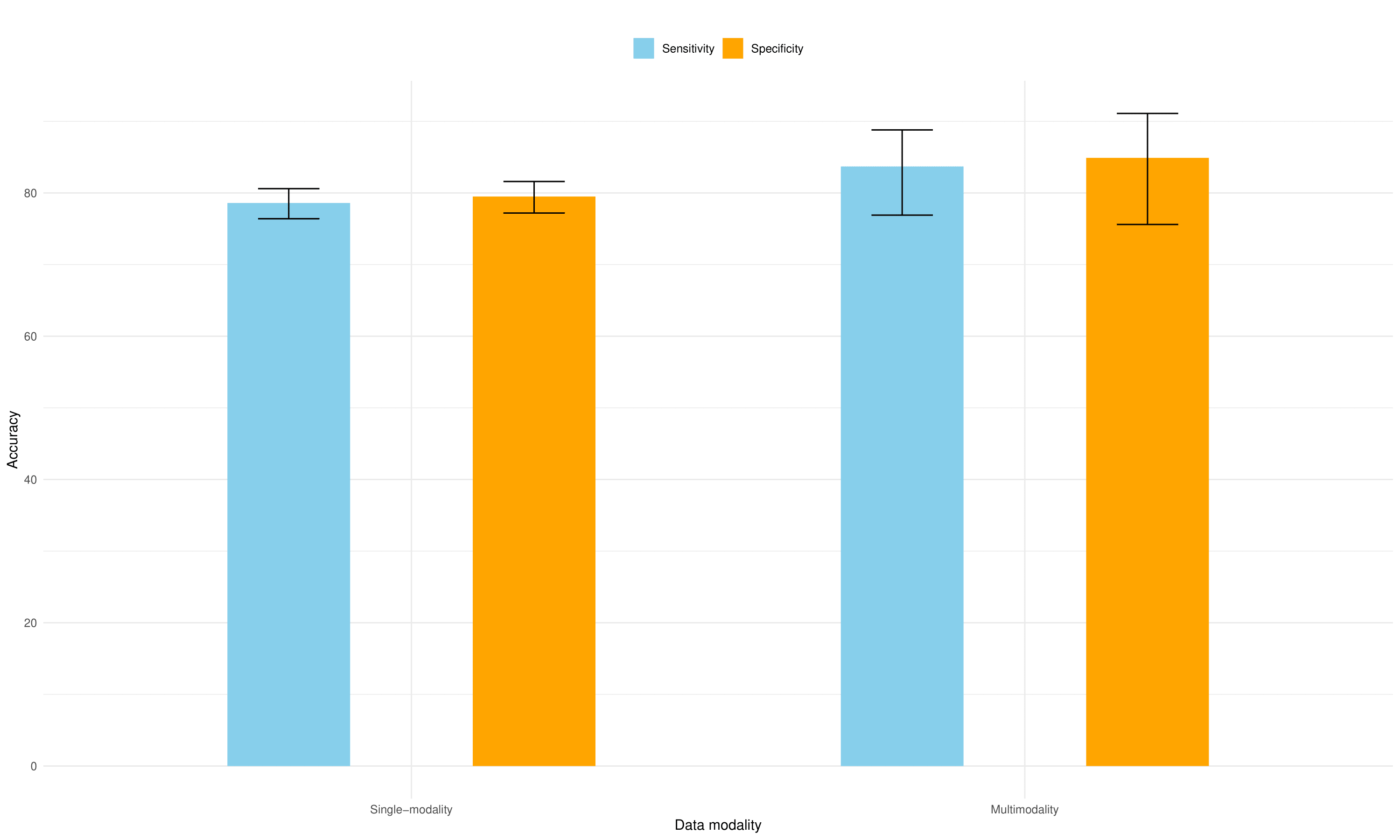
**

**Fig. S4 – Summary of classification accuracy for single-modality and multimodality data.**

**
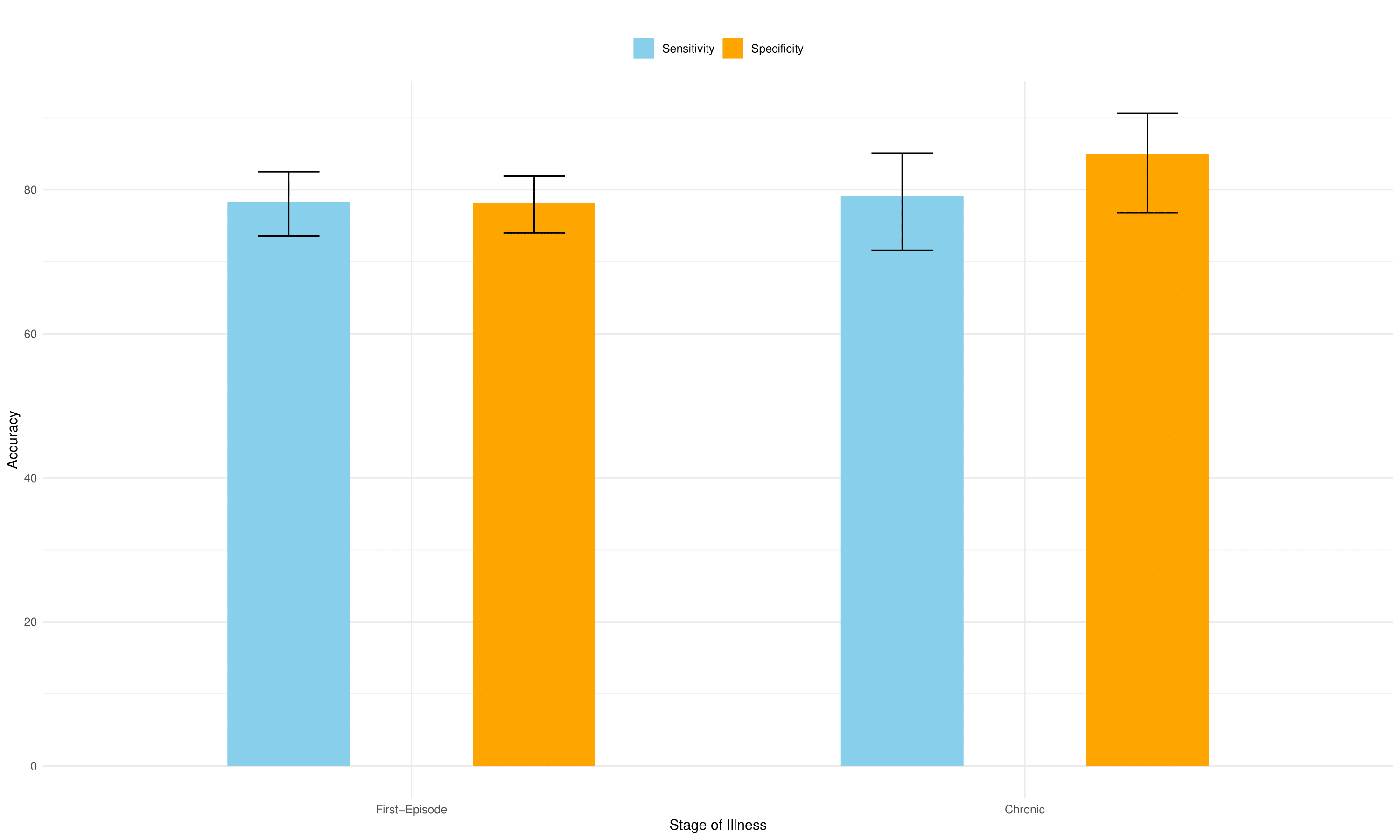
**

**Fig. S5 – Summary of classification accuracy for first-episode and chronic patients diagnosed with SSD**

**Tab. S11 – Meta-regressions results**

|  | **Sensitivity** | | | | | **False Positive Rate (1-Specificity)** | | | | |
| --- | --- | --- | --- | --- | --- | --- | --- | --- | --- | --- |
| **Covariates** | **Estimate** | **z** | **p** | **95%-CI** | | **Estimate** | **z** | **p** | **95%-CI** | |
| PANSS positive | 0.051 | 2.815 | **0.005*** | 0.016 | 0.087 | -0.064 | -4.025 | **<0.001*** | -0.096 | -0.033 |
| PANSS negative | 0.038 | 1.630 | 0.103 | -0.008 | 0.084 | -0.052 | -2.386 | **0.017*** | -0.094 | -0.009 |
| PANSS ratio | 1.077 | 2.177 | **0.030*** | 0.107 | 2.047 | -1.228 | -2.646 | **0.008*** | -2.137 | -0.318 |
| Illness Duration (y) | 0.012 | 0.827 | 0.408 | -0.017 | 0.042 | -0.022 | -1.683 | 0.092 | -0.048 | 0.004 |
| Medication. (cpz-eq) | -0.001 | -1.896 | 0.058 | -0.002 | 0.000 | 0.001 | 1.431 | 0.152 | < -0.001 | 0.001 |
| GAF | -0.034 | -2.563 | **0.010*** | -0.059 | -0.008 | 0.007 | 0.260 | 0.795 | -0.045 | 0.059 |
| Education SSD (y) | -0.104 | -1.563 | 0.118 | -0.234 | 0.026 | 0.073 | 1.115 | 0.265 | -0.055 | 0.200 |
| Age at Onset | 0.010 | 0.175 | 0.861 | -0.102 | 0.122 | -0.117 | -2.151 | **0.031*** | -0.224 | -0.010 |
| Age SSD | 0.003 | 0.335 | 0.738 | -0.016 | 0.022 | -0.029 | -2.737 | **0.006*** | -0.049 | -0.008 |
| Age tot | 0.004 | 0.390 | 0.696 | -0.016 | 0.024 | -0.023 | -2.063 | **0.039*** | -0.045 | -0.001 |
| DUP | 0.273 | 2.030 | **0.042*** | 0.009 | 0.536 | -0.258 | -1.815 | 0.069 | -0.536 | 0.021 |
| Female ratio SSD | 0.160 | 0.446 | 0.656 | -0.543 | 0.863 | -0.140 | -0.339 | 0.735 | -0.952 | 0.672 |
| Female ratio tot | -0.211 | -0.547 | 0.584 | -0.966 | 0.545 | 0.089 | 0.199 | 0.843 | -0.787 | 0.964 |
| Diagnostic criteria (ICD) | -0.202 | -0.773 | 0.440 | -0.716 | 0.311 | 0.222 | 0.750 | 0.453 | -0.359 | 0.804 |
| DTI | -0.231 | -1.109 | 0.267 | -0.639 | 0.177 | 0.109 | 0.469 | 0.639 | -0.347 | 0.565 |
| sMRI | -0.157 | -1.201 | 0.230 | -0.413 | 0.099 | 0.199 | 1.386 | 0.166 | -0.083 | 0.481 |
| rs-fMRI | 0.079 | 0.624 | 0.533 | -0.170 | 0.328 | -0.190 | -1.337 | 0.166 | -0.459 | 0.079 |
| task-fMRI | 0.103 | 0.537 | 0.591 | -0.273 | 0.478 | 0.137 | 0.666 | 0.505 | -0.267 | 0.542 |
| Multimodal | 0.354 | 1.572 | 0.116 | -0.087 | 0.795 | -0.308 | -1.208 | 0.227 | -0.808 | 0.192 |
| Deep Learning | 0.431 | 2.371 | **0.018*** | 0.075 | 0.787 | -0.497 | -2.519 | **0.012*** | -0.885 | -0.110 |
| Single site | 0.210 | 1.272 | 0.203 | -0.114 | 0.534 | 0.027 | 0.147 | 0.883 | -0.334 | 0.388 |
| External Validation | -0.582 | -2.710 | **0.007*** | -1.004 | -0.161 | 0.391 | 1.613 | 0.107 | -0.084 | 0.865 |
| First-Episode | -0.138 | -0.468 | 0.640 | -0.715 | 0.439 | 0.408 | 1.451 | 0.147 | -0.143 | 0.959 |
| Scan (3T) | -0.004 | -0.023 | 0.982 | -0.323 | 0.316 | -0.194 | -1.086 | 0.277 | -0.543 | 0.156 |
| Inpatients | -0.306 | -1.027 | 0.304 | -0.890 | 0.278 | 0.310 | 1.000 | 0.317 | -0.298 | 0.919 |
| Sample Size | < -0.001 | -0.369 | 0.712 | -0.001 | 0.000 | < -0.001 | -1.037 | 0.300 | -0.001 | < 0.001 |
| Algorithms: |  | | | | | | | | | |
| CNN | 0.585 | 2.162 | **0.031*** | 0.055 | 1.115 | -0.311 | -1.055 | 0.292 | -0.888 | 0.267 |
| DNN | 0.503 | 1.520 | 0.129 | -0.146 | 1.151 | -0.301 | -0.840 | 0.401 | -1.003 | 0.401 |
| SVM | 0.112 | 0.909 | 0.363 | -0.129 | 0.352 | -0.192 | -1.430 | 0.153 | -0.454 | 0.071 |
| G BOOST | -0.340 | -1.233 | 0.217 | -0.879 | 0.200 | -0.031 | -0.098 | 0.922 | -0.655 | 0.592 |
| DT | -0.251 | -0.780 | 0.435 | -0.881 | 0.380 | 0.140 | 0.398 | 0.691 | -0.549 | 0.829 |
| LDA | -0.281 | -1.368 | 0.171 | -0.683 | 0.121 | 0.200 | 0.855 | 0.392 | -0.258 | 0.657 |
| LR | -0.616 | -2.879 | **0.004*** | -1.036 | -0.197 | 0.446 | 1.850 | 0.064 | -0.027 | 0.918 |
| Feature Selection: |  | | | | | | | | | |
| Filter Methods | 0.539 | 3.442 | **0.001*** | 0.232 | 0.846 | -0.354 | -2.046 | **0.041*** | -0.694 | 0.015 |
| Embedded Methods | 0.291 | 1.419 | 0.156 | -0.111 | 0.693 | -0.280 | -1.248 | 0.212 | -0.719 | 0.160 |
| Multimodal | 0.312 | 1.713 | 0.087 | -0.045 | 0.668 | -0.355 | -1.772 | 0.076 | -0.748 | 0.038 |
| Wrappers | -0.124 | -0.364 | 0.716 | -0.795 | 0.546 | 0.116 | 0.302 | 0.763 | -0.637 | 0.869 |
| A priori | -0.321 | -1.619 | 0.105 | -0.709 | 0.068 | 0.374 | 1.691 | 0.091 | -0.059 | 0.807 |
| No Feature Selection | -0.419 | -3.262 | **0.001*** | -0.671 | -0.167 | 0.354 | 2.455 | **0.014*** | 0.071 | 0.637 |

**Fig. S6 – Moderation effect of PANSS positive score on sensitivity (left) and specificity (right).**

**Fig. S7 – Moderation effect of PANSS negative score on specificity.**

**Fig. S8 – Moderation effect of PANSS positive-to-negative ratio on sensitivity (left) and specificity (right).**

**
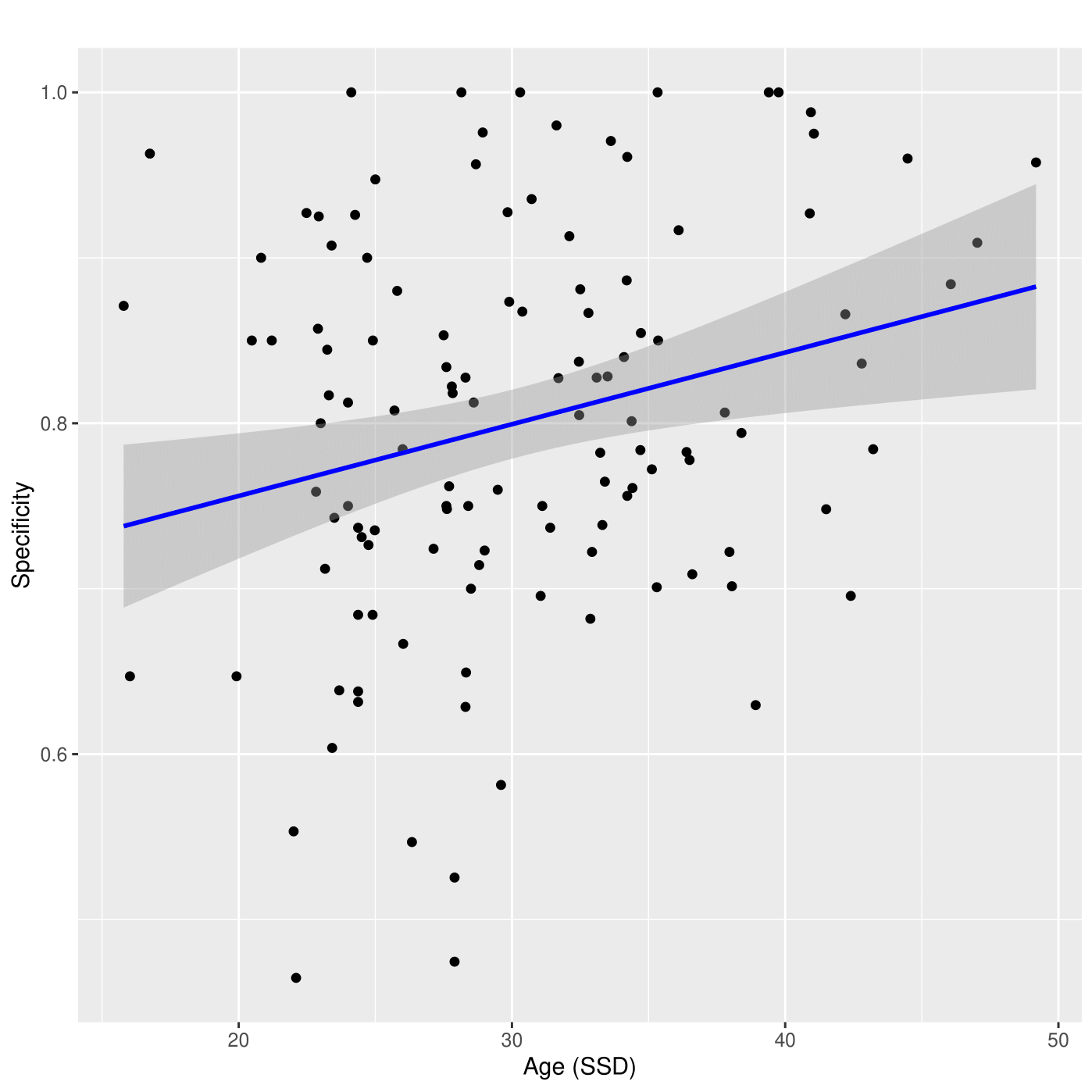

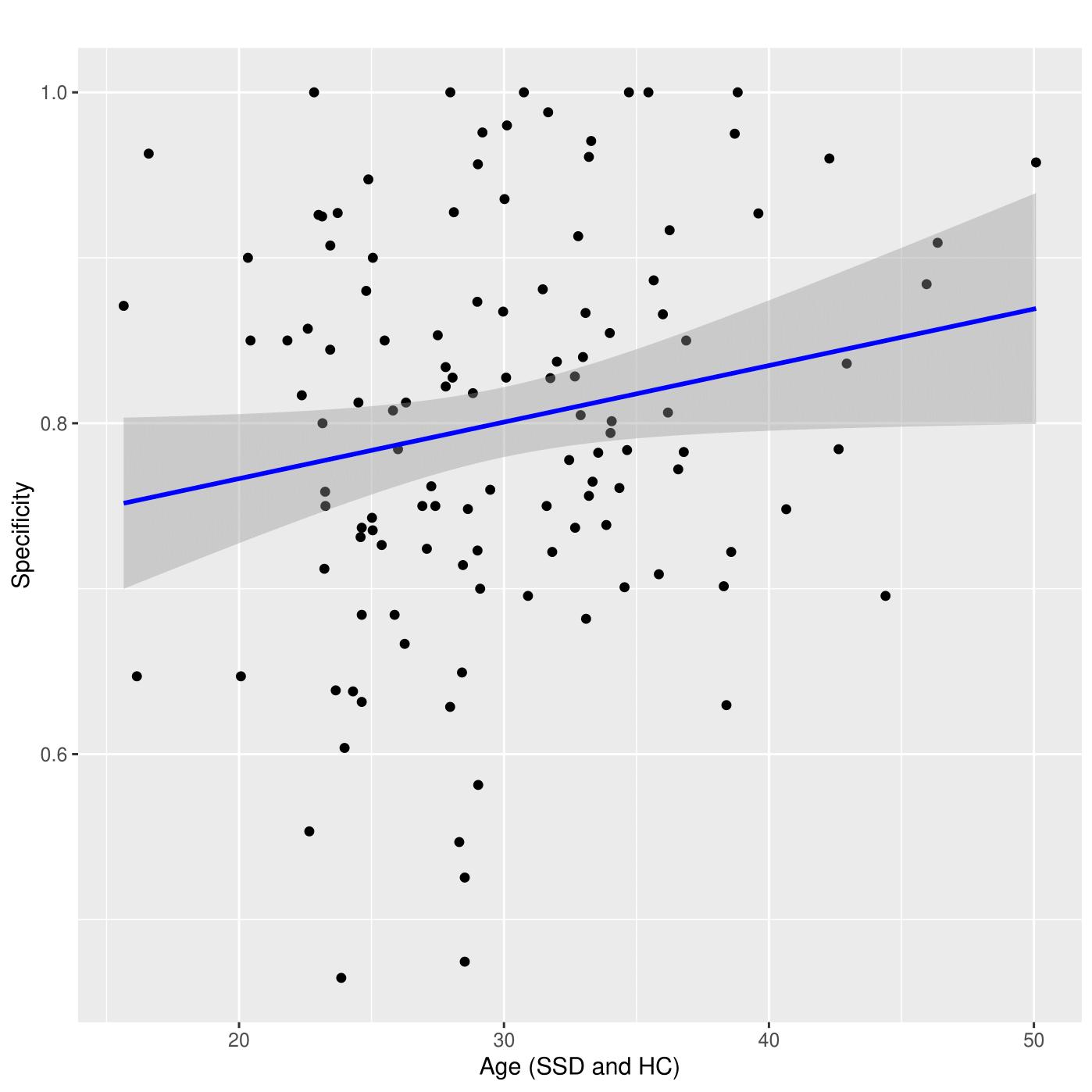
**

**Fig. S9 – Moderation effect of age of SSD (left) and of age of the whole sample (right) positive on specificity.**

**Fig. S10 – Moderation effect of age of onset on specificity.**

**Fig. S11 – Moderation effect of DUP on sensitivity. Fig. S12 – Moderation effect of GAF score on sensitivity .
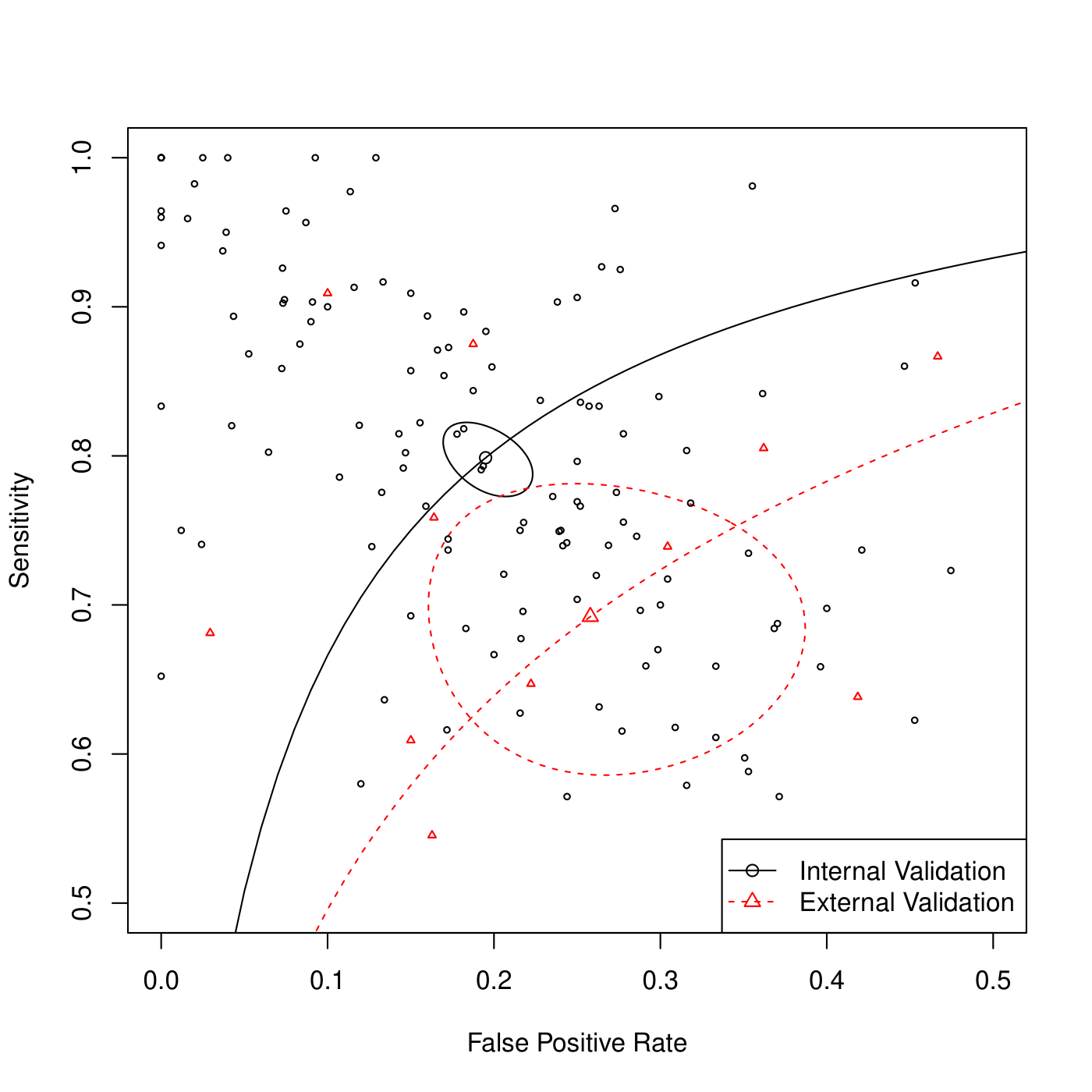
**

**Fig. S13 – Comparison of Summary Receiver Operating Characteristic (SROC) curves between Fig. S14 – Comparison of Summary Receiver Operating Characteristic (SROC) curves between studies employing internal and external validation results. deep learning methods and those that did not.**

**
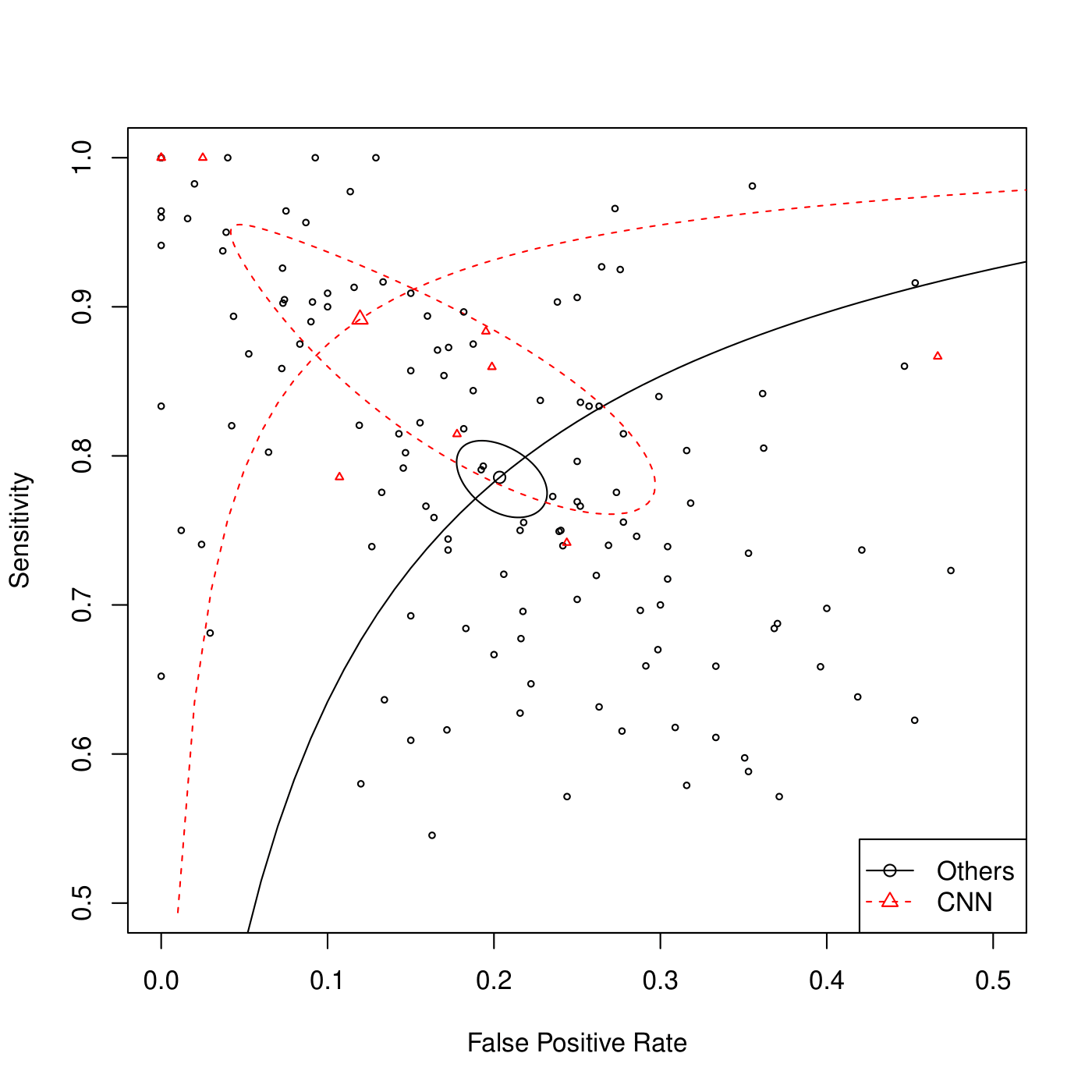

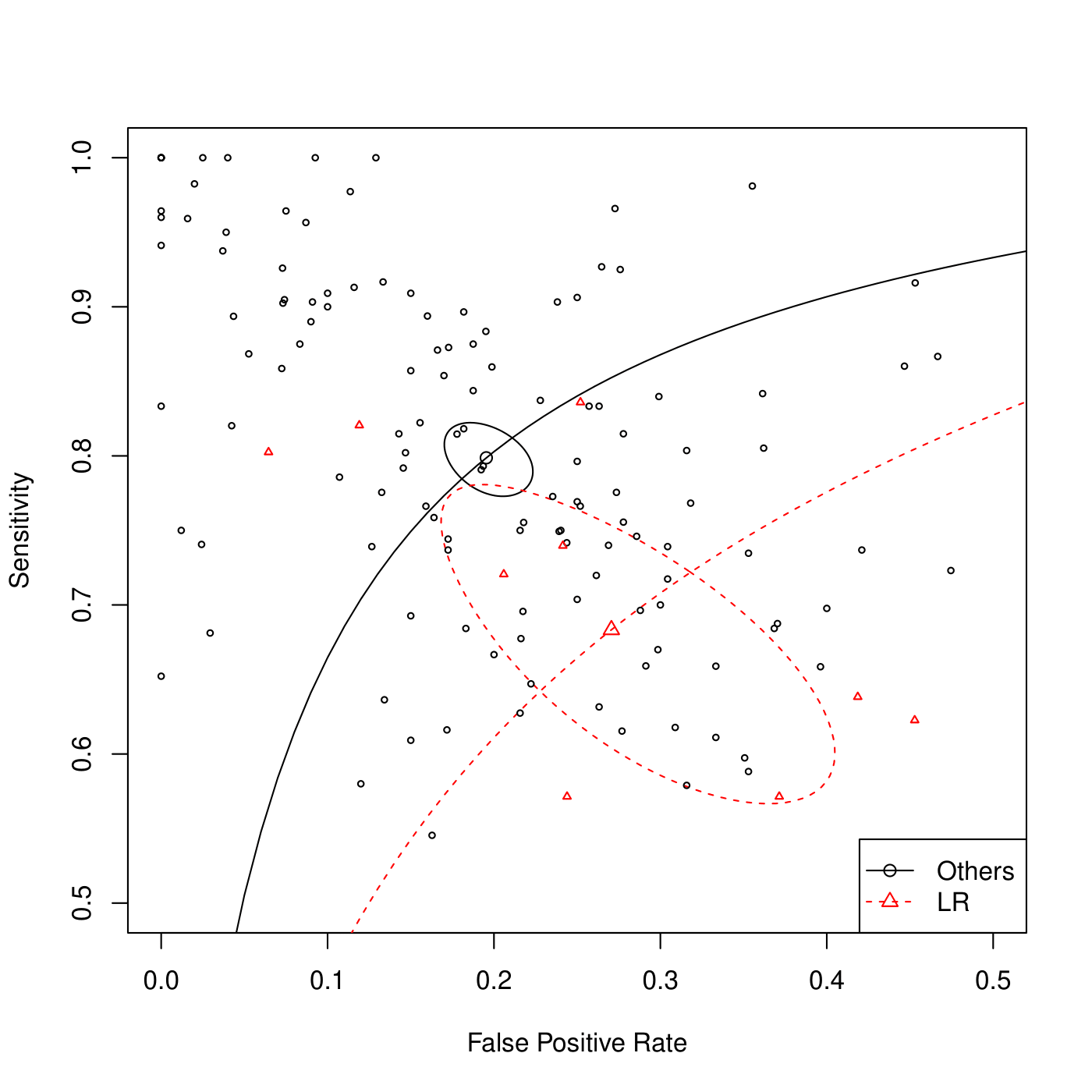
**

**Fig. S15 – Comparison of Summary Receiver Operating Characteristic (SROC) curves Fig. S16 – Comparison of Summary Receiver Operating Characteristic (SROC) curves between LR and other**

**between CNN and other algorithms. algorithms.**

**
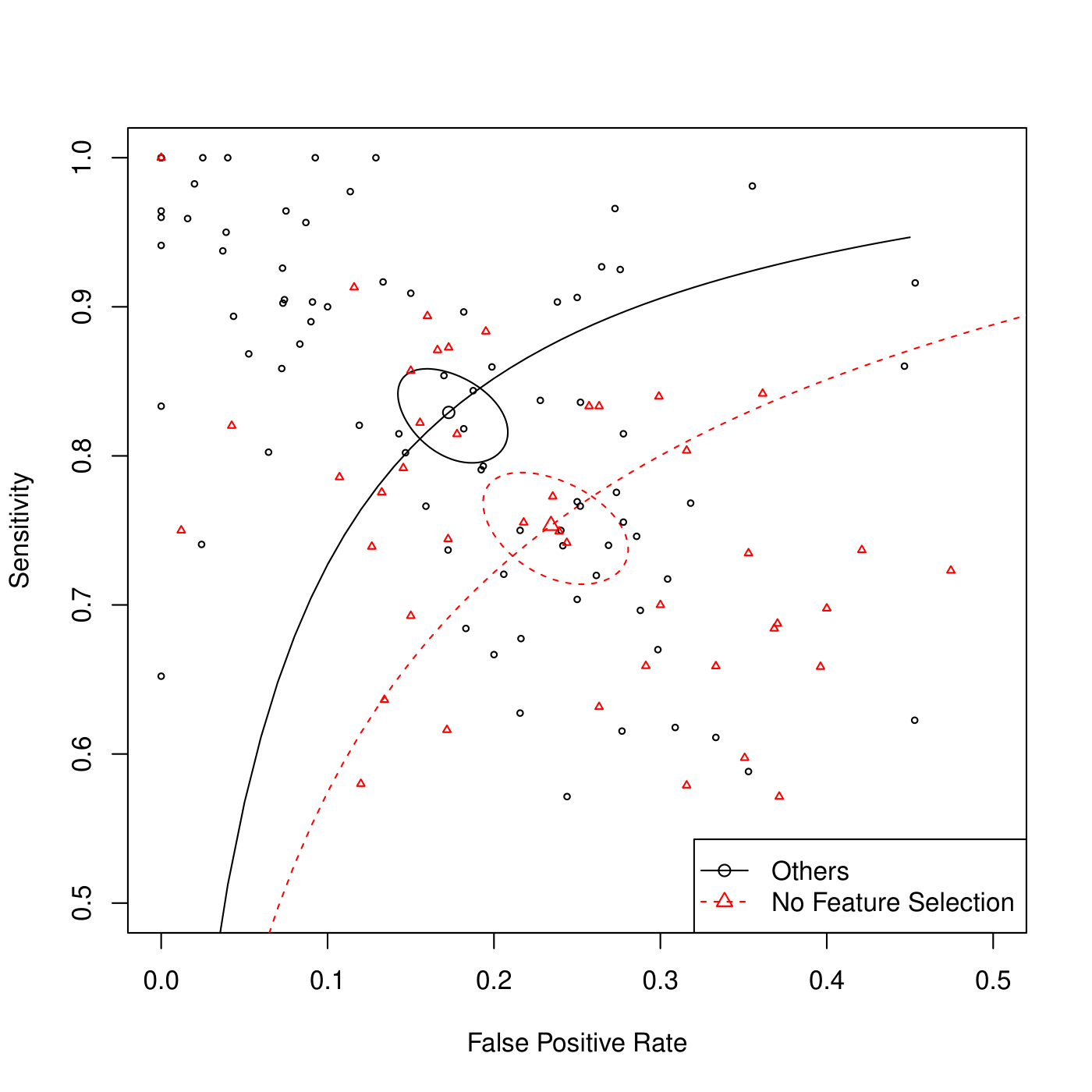

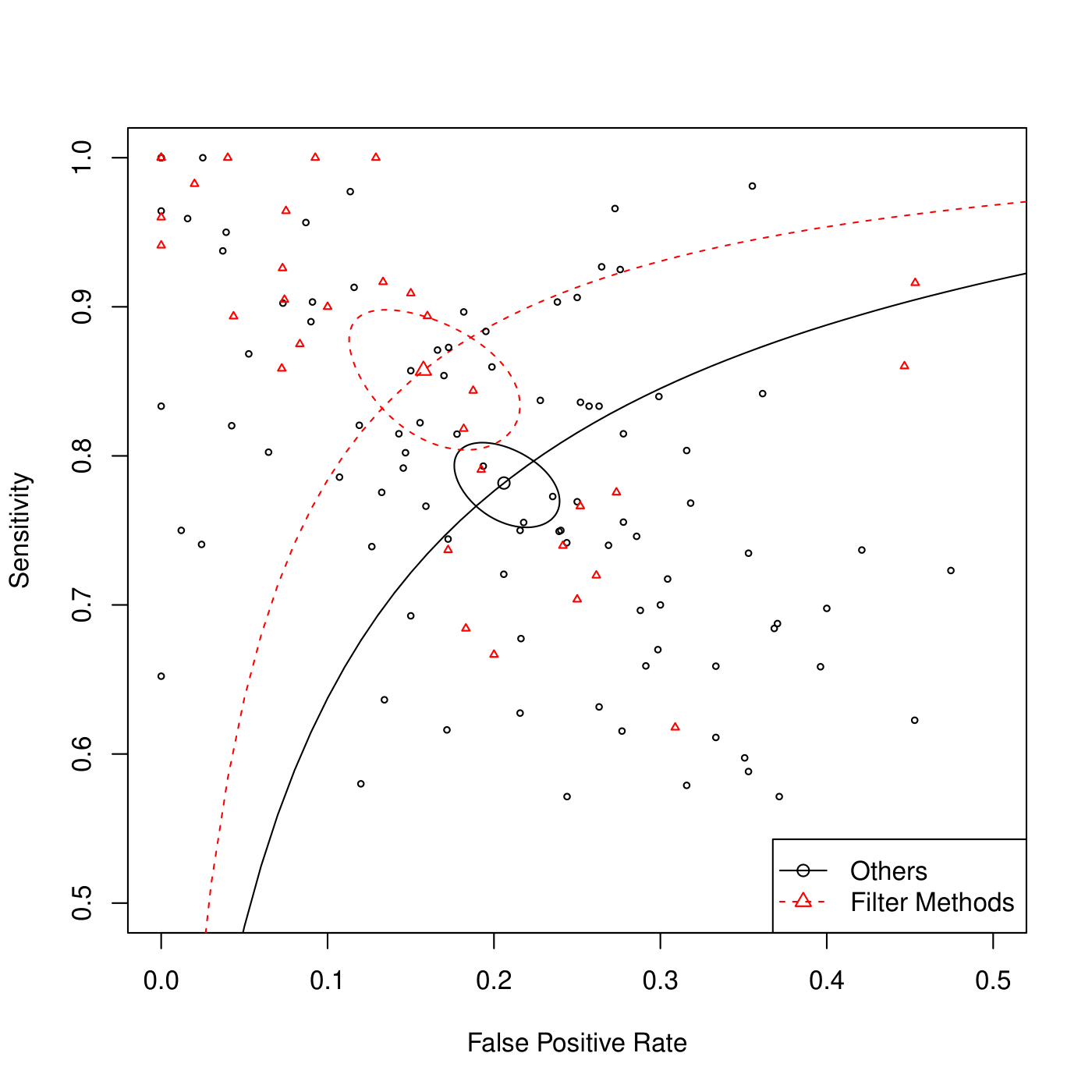
**

**Fig. S17 – Comparison of Summary Receiver Operating Characteristic (SROC) curves between Fig. S18 – Comparison of Summary Receiver Operating Characteristic (SROC) curves between studies employing studies employing any methods for feature selection and those that did not. filter methods for feature selection and those that did not.**

**
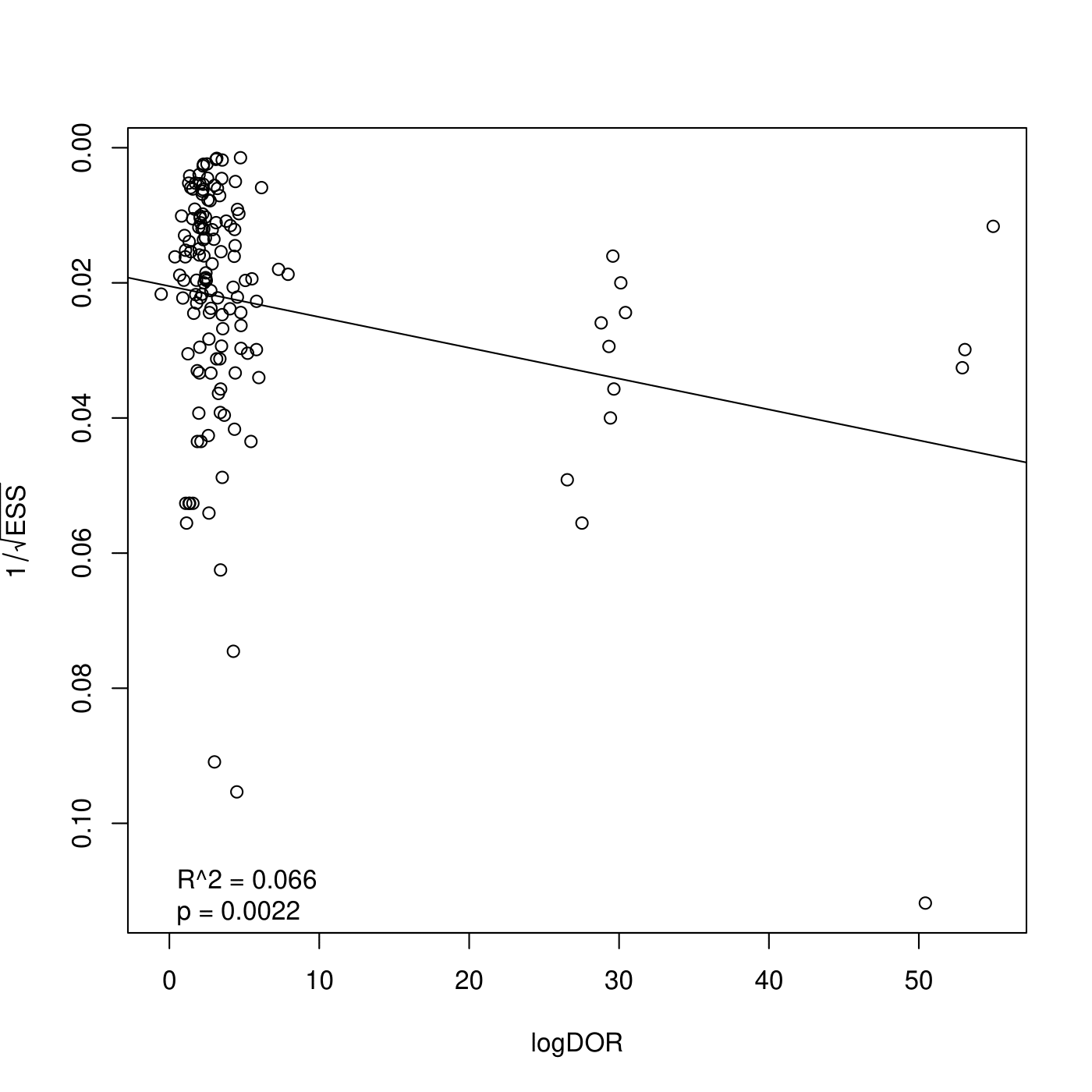
**

**Fig. S19 - Funnel Plot for Publication Bias**

**
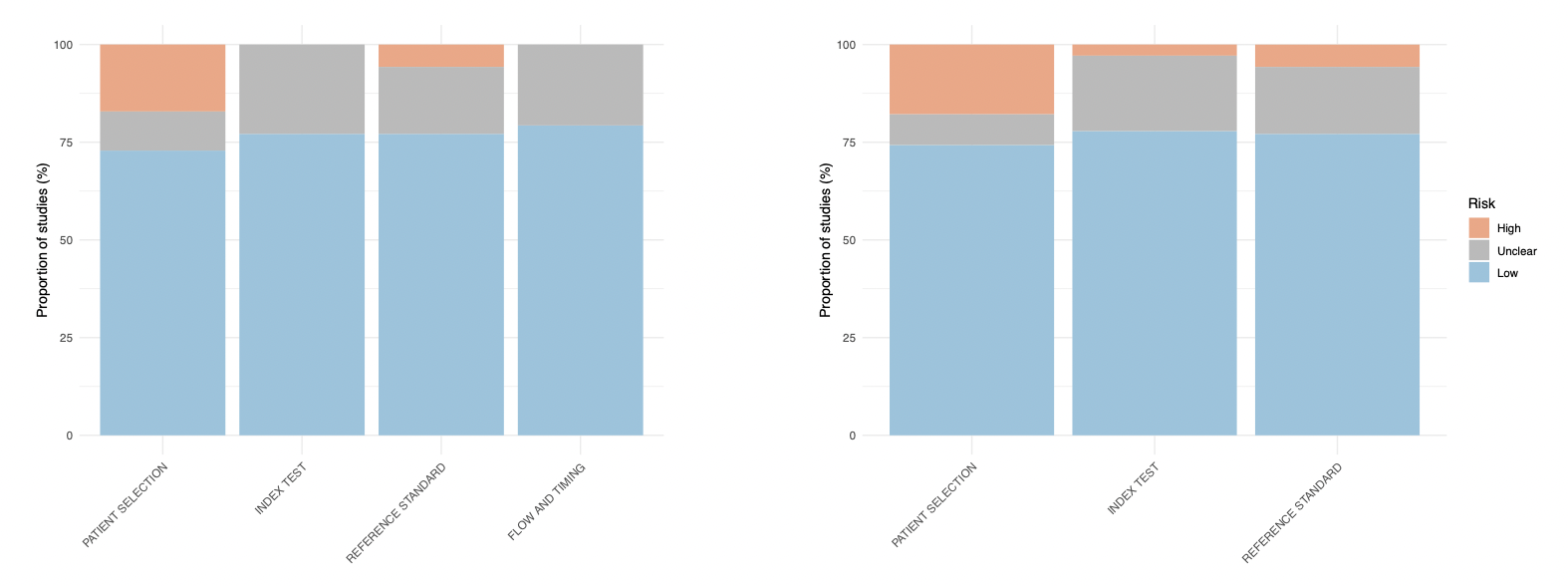
**

**Fig. S20 - Results of the assessment of the risk of bias (left) and of the applicability (right) of the studies included in the meta-analysis following the QUADAS-2 guidelines.**
